# Adolescent alcohol exposure persistently alters orbitofrontal cortical encoding of Pavlovian conditional stimulus components in female rats

**DOI:** 10.1038/s41598-024-64036-1

**Published:** 2024-06-14

**Authors:** Jose A. Pochapski, Alexander Gómez-A, Sierra J. Stringfield, Hannah Jaggers, Charlotte A. Boettiger, Claudio Da Cunha, Donita L. Robinson

**Affiliations:** 1 Laboratorio de Fisiologia e Farmacologia do Sistema Nervoso Central, Department of Pharmacology, Universidade Federal do Parana, Curitiba, PR Brazil; 2 Department of Biochemistry, Universidade Federal do Parana, Curitiba, PR Brazil; 3 Bowles Center for Alcohol Studies, University of North Carolina at Chapel Hill, Chapel Hill, NC USA; 4 Department of Psychiatry, University of Pittsburgh, Pittsburgh, PA USA; 5 Department of Psychology and Neuroscience, University of North Carolina at Chapel Hill, Chapel Hill, NC USA; 6 Department of Psychiatry, University of North Carolina at Chapel Hill, Chapel Hill, NC USA

**Keywords:** Reward, Motivation, Neurophysiology

## Abstract

Exposure to alcohol during adolescence impacts cortical and limbic brain regions undergoing maturation. In rodent models, long-term effects on behavior and neurophysiology have been described after adolescent intermittent ethanol (AIE), especially in males. We hypothesized that AIE in female rats increases conditional approach to a reward-predictive cue and corresponding neuronal activity in the orbitofrontal cortex (OFC) and nucleus accumbens (NAc). We evaluated behavior and neuronal firing after AIE (5 g/kg intragastric) or water (CON) in adult female rats. Both AIE and CON groups expressed a ST phenotype, and AIE marginally increased sign-tracking (ST) and decreased goal-tracking (GT) metrics. NAc neurons exhibited phasic firing patterns to the conditional stimulus (CS), with no differences between groups. In contrast, neuronal firing in the OFC of AIE animals was greater at CS onset and offset than in CON animals. During reward omission, OFC responses to CS offset normalized to CON levels, but enhanced OFC firing to CS onset persisted in AIE. We suggest that the enhanced OFC neural activity observed in AIE rats to the CS could contribute to behavioral inflexibility. Ultimately, AIE persistently impacts the neurocircuitry of reward-motivated behavior in female rats.

## Introduction

Adolescence is a critical period for executive function development in the brain^1,2^. This period is commonly marked by high engagement in novelty-seeking, risk-taking behavior and social interactions^2,3^. These behaviors are linked to limitations in “top-down control” due to developmental characteristics of cortical and limbic brain regions during adolescence^4,5^. Subcortical limbic regions important for reward processing and motivation, such as the nucleus accumbens (NAc) and ventral tegmental area, mature earlier than the prefrontal cortex, which is essential for executive function^4,5^. Activation of limbic regions along with a lack of cortical control can facilitate the use of alcohol and other drugs in adolescents^2^. Adolescents are more sensitive to alcohol-induced stimulation, social facilitation, and behavioral disinhibition, and less sensitive to sedative and motor impairments^1,2^. A pattern of binge-drinking, where individuals drink high amounts of alcohol in a short period of time (five or more drinks in a session for men and 4 or more drinks for women) is often seen in adolescents^6^. Binge-drinking episodes are often associated with adverse consequences, including increased risk-taking and impaired decision-making^7^.

Preclinical rodent studies demonstrate that adolescent intermittent ethanol (AIE) exposure induces impairments in memory^8^ and cognitive flexibility^9–11^, and increases alcohol self-administration^12,13^. Interestingly, Broadwater et al.^14^ showed that AIE reduced resting-state functional connectivity between the orbitofrontal cortex (OFC) and the NAc, important regions for reward-related information processing. Moreover, Gómez-A et al.^11^ linked AIE-induced reductions in frontostriatal functional connectivity to deficits in behavioral flexibility. Neuronal activity in the OFC is necessary for encoding the association between a stimulus and an outcome, as well as the outcome’s motivational value^15^, resulting in key modulation of reward-seeking behaviors^16^. NAc function is implicated in associative learning and reward-motivated behavior^17^, and increases in NAc neuronal firing can invigorate reward-seeking and induce reward-approach behavior^18^. Studies have described AIE-induced changes in OFC and NAc that can lead to impairment in reward processing and decision-making^2,9,11,19,20^. Importantly, AIE effects are often long-lasting and persist into adulthood, making AIE exposure a potential risk factor in the development of alcohol use disorder in adulthood^20–22^.

Pavlovian conditioned approach (PCA) paradigms can be used to evaluate the long-lasting effects of AIE exposure on reward-related behavior. In this task, the repeated presentation of a cue light (conditional stimulus, CS) and reward delivery (unconditional stimulus, US) lead animals to learn the predictive association and the temporal relationship between those events^23^. In many cases, the animal’s behavior can be classified into two distinct phenotypes: sign-tracking and goal-tracking. Characteristically, after the CS onset but before US delivery, sign-tracking (ST) animals will approach and interact with the CS, while goal-tracking (GT) animals will approach the site of reward delivery. In sign-tracking animals, the CS may acquire incentive salience^24,25^. Sign-tracking behavior is thought to be associated with impulsive behaviors and addiction vulnerability, and studies show that alcohol exposure during adolescence can increase the expression of sign-tracking behavior^20^. Moreover, Madayag et al.^26^ found that AIE exposure increased sign-tracking and reduced goal-tracking in both males and females compared to controls, and female rats presented higher levels of sign-tracking behavior than males.

During PCA, different neuronal substrates can modulate the expression of behavioral responses. Previous studies report that sign- and goal-tracking behavioral expression may result in part from different learning mechanisms encoded by the NAc^27^. The OFC role in Pavlovian approach was also previously demonstrated^27–29^. Specifically, electrophysiological recordings during PCA demonstrate robust OFC neuronal activity to predictive CS and conditioned approach^28^. Also, previous studies found increased c-fos mRNA expression to a CS in the OFC^27^, and that OFC inactivation decreases sign-tracking behavior^28^. However, the roles of OFC and NAc in PCA and AIE effects on these brain regions have almost exclusively been studied in male as opposed to female rats. The present study adds to the literature by evaluating the behavioral and neuronal effects of AIE exposure on conditioned behavior in female rats. We aimed to monitor OFC and NAc activity as part of a broader circuit, rather than to focus on direct connectivity; thus, contralateral, instead of ipsilateral, electrophysiological recordings in OFC and NAc were performed. We hypothesized that AIE would shift Pavlovian approach toward sign-tracking and away from goal-tracking, and that these changes would be associated with enhanced OFC and NAc neuronal firing activity during the CS-US presentation.

## Results

### Behavioral responses during PCA baseline and reward omission session in sign-tracking animals

**Figure 1:**
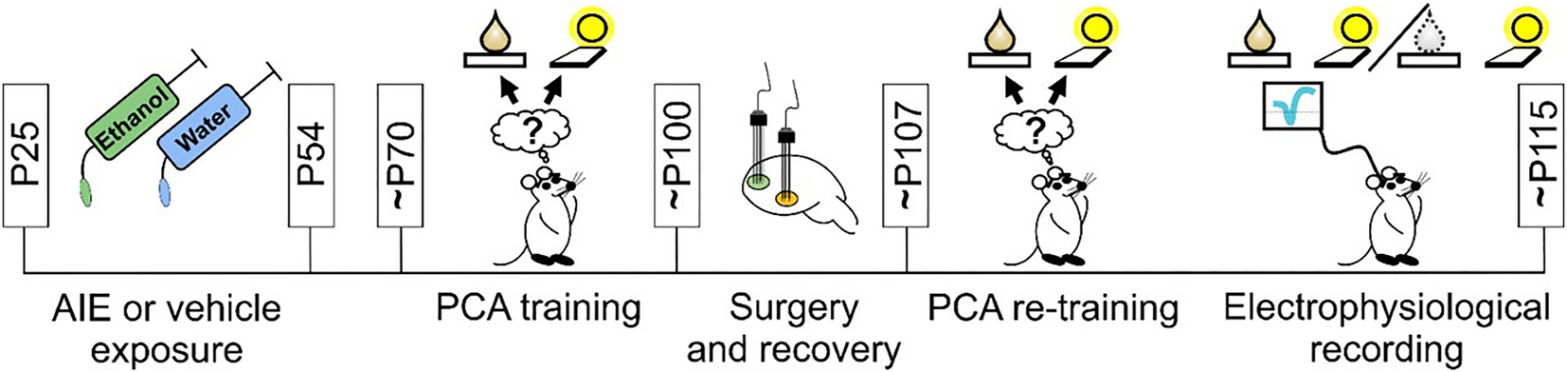
Experimental timeline. Female Sprague–Dawley rats received ethanol administration across the adolescent period, from postnatal day (P) 25 to P54 on a 2-days-on / 2-days-off schedule. After this period no additional ethanol was administered. Starting around P70, a total of 20 Pavlovian condition approach (PCA) training sessions were performed. Each PCA session consisted of 15 trials where a 30-s (s) conditioned stimulus (CS, cue light/lever extension) was presented, followed by the US presentation (0.1 ml of a 20% sucrose solution) immediately after the CS offset. After the training phase, a stereotaxic surgery procedure was performed for electrode array implantation in the OFC and NAc, followed by 7 days of recovery. At least 5 additional PCA sessions occurred after the surgery recovery in order to habituate the animals to the electrophysiological recording procedure. Next, to evaluate possible AIE-induced effects on neuronal activity, single-unit recordings in OFC and NAc were performed during a regular PCA session (baseline) followed by a reward omission session. During the reward omission session, all 15 trials were performed similarly to the baseline session, with one important exception: after the 30-s CS, no reward solution was delivered in the reward receptacle. In the figure, the colored drop illustrates the US presentation during PCA session and the gray dotted drop represents the reward omission session. For more details, see methods.

**Figure 2:**
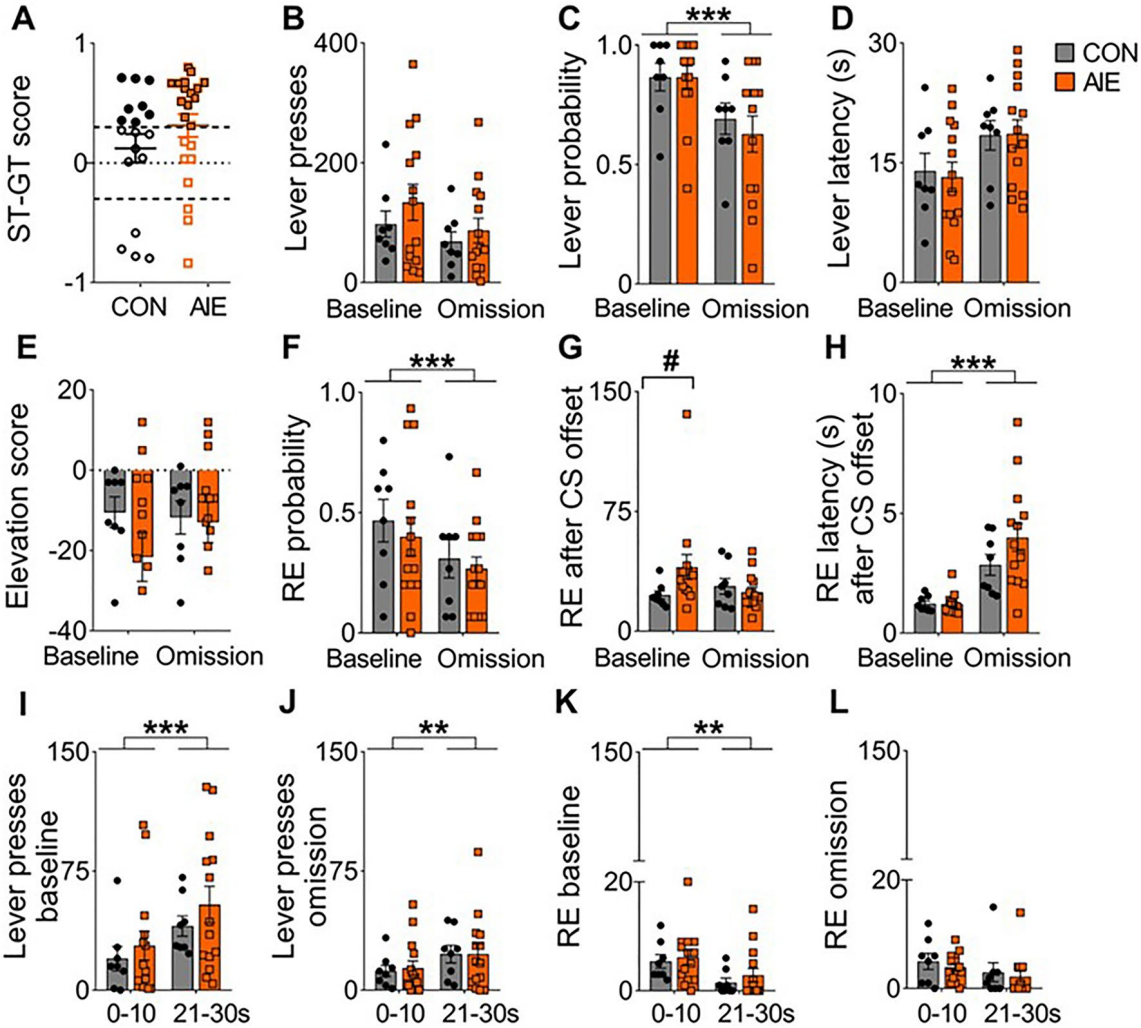
Little effect of AIE exposure was observed on sign-tracking and goal-tracking behavioral responses in sign-tracking rats. (**A**) Using a composite sign-tracking/goal-tracking (ST-GT) score, rats were categorized as goal-trackers (CON n = 4, AIE n = 3), sign-trackers (CON n = 8, AIE n = 14), and intermediate (CON n = 6, AIE n = 5), see methods. Results here focused on sign-trackers (filled symbols). (**B**) Total number of lever presses. No significant differences were observed. (**C**) Lever press probability. A marginal exposure effect and a significant session effect were observed. However, no interaction between the factors was observed. (**D**). Latency to perform the first lever press. No significant differences were observed. (**E**). Receptacle elevation score. No significant differences were observed. (**F**). Probability of receptacle entry during the 30 s CS period. A marginal exposure effect and a significant effect of session were observed. However, no interaction between the factors was observed. (**G**). Number of receptacle entries (RE) 10 s after CS offset. A significant interaction of exposure by session was observed. Post-hoc analysis demonstrated a higher number of RE during the baseline session in the AIE versus CON groups. (**H**). Receptacle entry after CS offset. A significant effect of session, but no exposure or interaction between the factors were observed. (**I-L**). Averaged number of lever presses (**I-J**) and receptacle entries (**K-L**) during the first 10 s after CS onset (0–10 s) and during the 10 s before CS offset (21–30 s). (**I**). Analysis during baseline session demonstrated a significant effect of time. No treatment effect or interaction were observed. (**J**). Similarly, during the omission session only an effect of time was observed. (**K**). During the baseline session, only a significant effect of time was observed. (**L**). No significant differences were observed during the omission session. Data are expressed as mean ± SEM. The symbols in black circles for CON (n = 8) and orange squares for AIE (n = 14) represent individual subjects' data for sign-tracking rats. *** Main effect of session (*P* < 0.001). # *P* ≤ 0.05 group difference after Sidak’s post-hoc comparison. For statistical details see Table 1.

Female rats were initially trained on a PCA paradigm where the CS onset (cue light) was presented concurrent with a lever extension (below the light stimulus location) for 30 s. After this period, the CS offset event (light offset) was followed by the lever retraction, and the 20% sucrose reward (US) was delivered (Fig. 1). A total of 25 PCA training sessions were performed. Next, a stereotaxic surgery was performed where multi-array electrodes were implanted in OFC and NAc. Following a recovery period, rats resumed the PCA training. After performing a baseline PCA session, rats were categorized according to the predominant phenotype (sign-tracking, goal-tracking or intermediate, see Methods). Based on a composite ST-GT score, we found a predominance of sign-trackers (AIE:14 and CON: 8 rats) in both groups when compared to goal-trackers and intermediate rats (Fig. 2 A). As both behavioral phenotype and AIE exposure could contribute to differences in neuronal firing patterns, and because we primarily aimed to evaluate the consequences of AIE on reward processing, the remaining analyses focused exclusively on the sign-tracker rats. By equating the behavioral phenotype, we could more confidently attribute group differences in neuronal firing patterns to AIE exposure.

We next assessed specific metrics of conditioned approach only in the sign-tracking subset of rats, comparing behavioral responses between groups and sessions. Behavioral analysis on sign-tracking metrics (total number, latency and probability of lever presses) and goal-tracking metrics (receptacle elevation score, latency and probability of receptacle entry during the 30 s CS period) were performed. As we used a long CS period (30 s), and CS onset and CS offset events can indicate different aspects of the task, we also accounted for lever presses and receptacle entries surrounding the CS onset and CS offset events. Analysis of sign-tracking behavior demonstrated similar results between groups, with no significant main effect of exposure (P’s > 0.05; Fig. 2B–D and Table 1) and only session effects on lever probability. Moreover, while the total number and the latency to press the lever did not significantly differ by session, the probability of pressing the lever during each trial decreased through reward omission (*P* < 0.001; Fig. 2C). Analysis across each session also indicated that the CON group, but not the AIE group, significantly increased the latency to press the lever as the baseline session progressed (*P* ≤ 0.05; Supplemental Fig. S1 D) and both groups increased the latency across the omission session (*P* < 0.001; Supplemental Fig. S1 E and Supplemental Table S1).

**Table 1:** Statistical analysis results of behavioral data from Pavlovian conditioned approach baseline and reward omission session on sign-tracking female rats.

I Behavioral parameters					
Variable	Effect	Exp(B)	P	Confidence interval Lower Upper	
Lever presses	Exposure	1.267	0.56	0.561	2.859
	Session	1.426	0.43	0.591	3.443
	Exp*Session	0.885	0.88	0.376	3.110
Lever latency	Exposure	0.948	0.98	0.005	190.2
	Session	0.009	0.12	0.001	3.425
	Exp*Session	0.585	0.88	0.000	1055
Lever probability	Exposure	0.910	***0.08***	0.818	1.012
	Session	1.255	** < 0.001**	1.123	1.404
	Exp*Session	1.099	0.19	0.954	1.266
Receptacle probability	Exposure	0.870	***0.08***	0.741	1.021
	Session	1.514	** < 0.001**	1.289	1.778
	Exp*Session	0.985	0.88	0.801	1.212
RE 10 s after CS offset	Exposure	0.872	0.138	0.586	1.300
	Session	0.804	0.352	0.547	1.180
	Exp*Session	2.041	**0.016**	1.141	3.653
RE latency after CS offset	Exposure	1.529	0.230	-1,53	0,391
	Session	32.17	** < 0.001**	-3,00	-1,391
	Exp*Session	2.226	0.151	-1,53	0,391
**Variable**	**Effect**	**F**	***P***	**Df**	
Elevation score	Exposure	0.875	0.360	1	__
	Session	0.692	0.415		
	Exp*Session	1.240	0.278		
LP 10 s after CS onset and 10 s before CS offset on baseline session	Exposure	0.660	0.426	1	
	Time	13.24	**0.001**		
	Exp*Time	0.176	0.678		
LP 10 s after CS onset and 10 s before CS offset on omission session	Exposure	0.014	0.906	1	
	Time	8.221	**0.009**		
	Exp*Time	0.073	0.788		
RE 10 s after CS onset and 10 s before CS offset on baseline session	Exposure	0.508	0.484	1	
	Time	8.853	**0.007**		
	Exp*Time	0.059	0.809		
RE 10 s after CS onset and 10 s before CS offset on omission session	Exposure	0.579	0.455	1	
	Time	2.559	0.125		
	Exp*Time	0.015	0.903		

Behavioral analyses were conducted using a generalized linear model with a Poisson distribution and a log link function or using a two-way ANOVA depending on the data distribution. Group comparison on lever presses 10 s before CS offset were performed using Mann–Whitney U test or *t*-test depending on data distribution. Underlined bold type indicates significant differences (*p* values are described on the table). Italics bold type indicates marginal differences (see methods). CS (conditional stimulus); Exp (exposure); LP (lever presses); RE (receptacle entries).

Analysis of the goal-tracking behavior demonstrated that both CON and AIE-exposed rats presented a negative elevation score, indicating that they entered the receptacle less during the CS than in the 30 s prior to the CS, with no statistical difference between groups (P's > 0.05; Fig. 2E, Table 1). Similar results were observed for the total number of receptacle entries performed before CS onset and during CS onset (P’s > 0.05; Supplemental Fig. S1A,B; Supplemental Table S1). However, analysis on the receptacle entries after the CS offset demonstrated that AIE-exposed rats performed more entries on baseline session (*P* ≤ 0.05; Fig. 2G; Table 1), and both groups performed similarly in the omission session (*P* > 0.05). Metrics of probability and latency to enter the receptacle differed between baseline and omission sessions (*P* < 0.001 and *P* < 0.01, respectively), showing a decrease in the probability and an increase in the latency to enter the receptacle during CS period during the omission session. No effect of AIE exposure was observed on any of these metrics (*P* > 0.05; Fig. 2F and Supplemental Fig. S1 C; Table 1 and Supplemental Table S1, respectively). After CS offset, both groups showed similar increases in the latency to enter the reward receptacle on the omission session compared to the baseline session, and also across the omission session (P’s < 0.001; Fig. 2H and Supplemental Fig. S1 F,G; Table 1 and Supplemental Table S1), with no AIE effect observed (P’s > 0.05).

As our experiment used a 30-s CS presentation, we next asked whether behavioral responses changed across the CS presentation. In order to test this, lever and receptacle responses were compared during the initial and final 10 s of the CS. The last 10 s of the CS coincided with twice as many lever responses as the initial period (Fig. 2I; main effect of time, *P* < 0.001). This pattern remained consistent in the omission session (Fig. 2J; *P* < 0.001). Conversely, the maximum number of receptacle entries was observed during the 10 s after CS onset during the baseline session (Fig. 2K; main effect of time, *P* < 0.01), although no differences were observed during the omission session (Fig. 2L *P* > 0.05). No significant effect of AIE exposure or interaction were observed during these analyses (P's > 0.05; Table 1).

In summary, our results revealed a high expression of sign-tracking phenotype in both AIE and CON-exposed female rats. When considering only sign-tracking rats, conditioned approach behavior did not statistically differ between groups, although it was affected by withholding the US (omission).

### Single-unit recording during PCA baseline and reward-omission sessions

To evaluate whether AIE exposure altered neuronal activity in the OFC and NAc of sign-tracking animals, single-unit recordings were conducted during the baseline and reward-omission sessions. Histological analysis (Supplemental Fig. S2) confirmed that both groups presented similarly distributed electrode locations in the medial and lateral OFC (CON: 57; AIE: 93) and as intended, the majority of the NAc placements were located in the core subregion for both groups (CON: 55; AIE: 89). An average of 11.6 ± 2.1 and 8.1 ± 2.7 neurons were recorded from each rat (range 2–18 and 2–13 neurons) in CON and AIE groups, respectively. We monitored firing rates of OFC and NAc neurons at the CS onset and offset, lever presses, and receptacle entries. For the baseline session, neuronal firing was signal-averaged across the 15 trials of the baseline session, described below. For the omission session, our analysis focused on trials 6–15, after the rats experienced the first five trials with no reward and updated their behavioral response accordingly (Supplemental Fig. S3 C). No significant differences were observed in the whole session firing rate on OFC and NAc during the baseline and reward omission session (P’s > 0.05; for details see Supplemental Table S2).

### AIE exposure increased OFC neuronal activity in response to the CS but not conditioned approach

**Figure 3:**
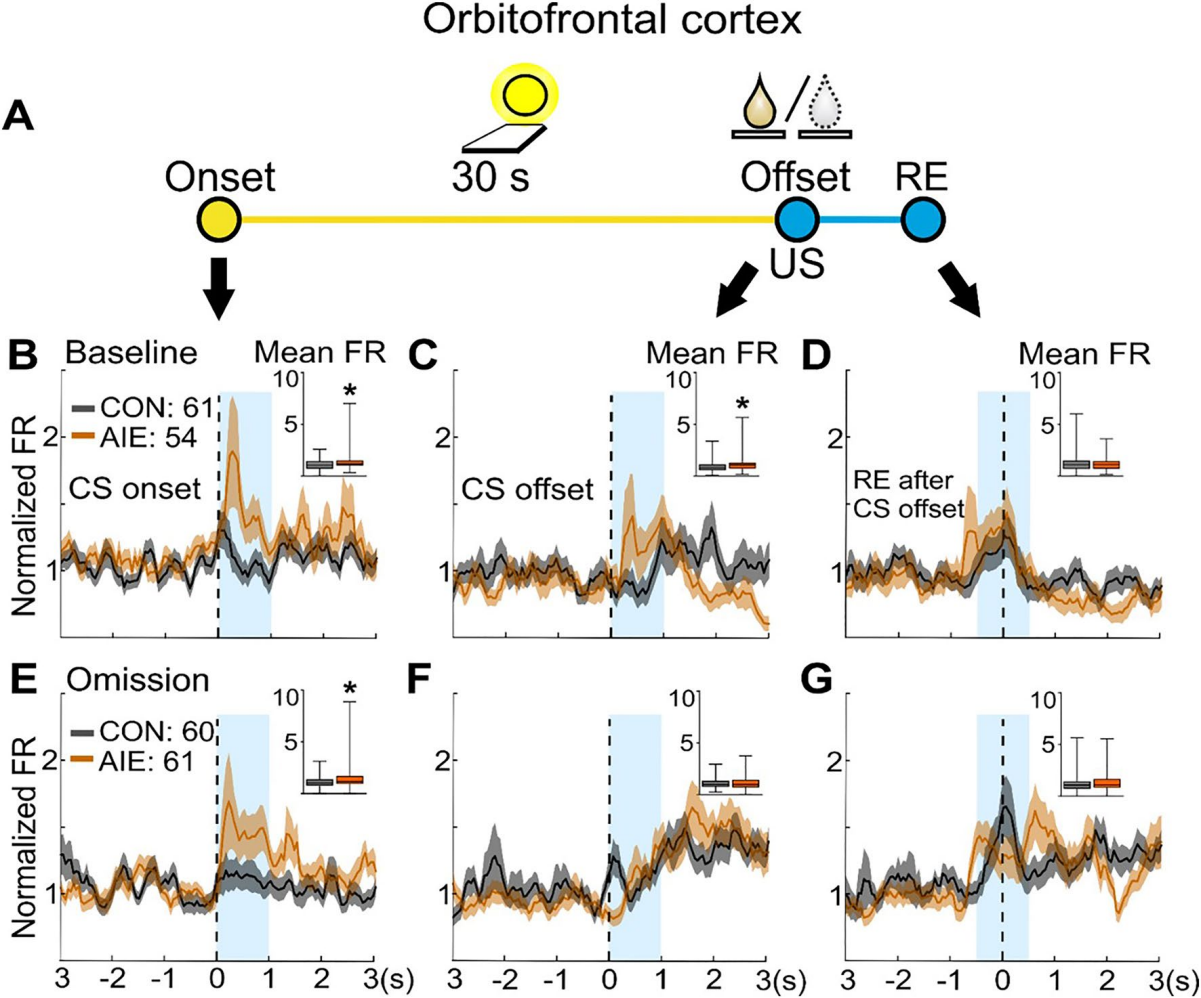
AIE exposure induced long-term changes in the OFC neuronal activity in response to CS onset and CS offset. Single-unit activity of OFC neurons was analyzed in sign-tracking rats during the baseline and omission sessions. (**A**). Illustrative representation of each trial during PCA baseline and omission sessions. In each trial, a 30-s conditioned stimulus (CS, cue light/lever extension) was presented. After the CS offset, the unconditioned stimulus (US, sucrose) was delivered during PCA baseline session. During the omission session, no US was delivered. Analyses were conducted on OFC single-unit population activity 1 s after CS onset and CS offset and 0.5 s before and after the receptacle entry performed after the CS offset (reward retrieval). (**B-D**). OFC population activity (average of all neurons) during baseline session. (**B**). Neurons recorded in AIE-exposed rats presented significantly higher population excitatory activity after CS onset than CON-exposed rats. (**C**). Analysis after CS offset also indicated a significantly higher OFC population activity in AIE-exposed compared to CON-exposed rats. (**D**). No AIE effect was observed on OFC activity during the first receptacle entry after CS offset. (**E–G**). OFC population activity during omission session. (**E**). After changes in the CS-US contingency a significantly higher excitatory activity after CS onset was still observed in OFC population activity in AIE-exposed group. (**F**). No AIE effect was observed on OFC population activity after CS offset during omission session. (**G**). Compared to the CON-exposed group, no AIE effect was observed on OFC activity during the first receptacle entry after CS offset. Neuronal population activity data are normalized to the whole session firing rate and presented as mean firing rate (line) ± SEM (shading) of all recorded neurons for CON (gray) and AIE (orange) groups. Number of recorded cells on OFC during baseline session: 61 cells in CON rats; 54 cells in AIE rats. Number of recorded cells on OFC during omission session: 60 cells in CON rats; 61 cells in AIE group. Inset graphs: box plots show the mean firing rate in the target window (blue box). * Significant group difference in mean firing rate (*P* ≤ 0.05). For statistical details, see Table 2.

**Figure 4:**
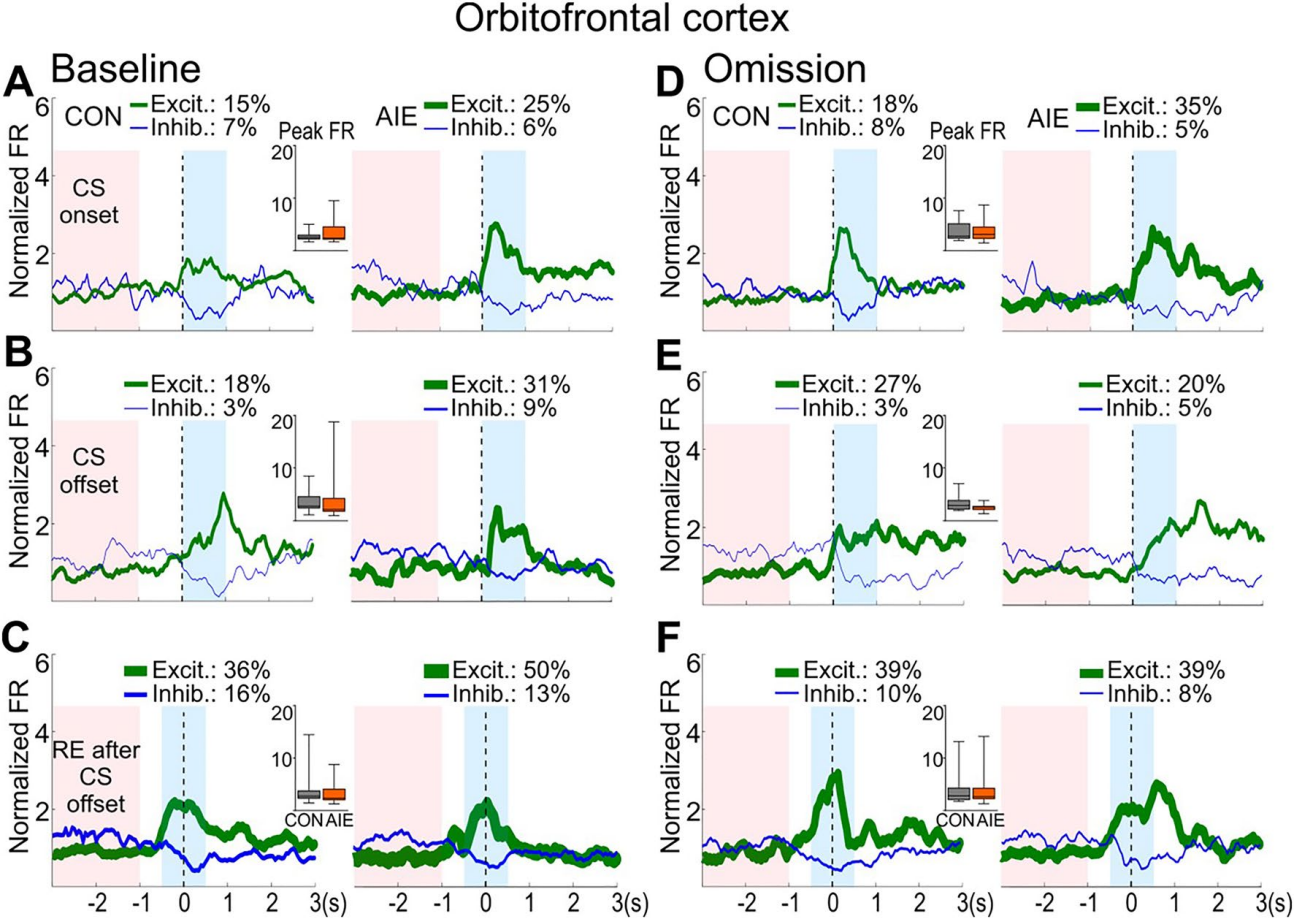
No AIE exposure effect was observed on the amplitude of the OFC phasic excitatory cells. Phasic neuronal activity during CS onset, CS offset and first receptacle entry after CS offset from OFC recorded neurons during the PCA baseline and omission sessions. Firing activity for neurons that showed phasic excitatory activity (in green) and phasic inhibitory activity (in blue) are shown in each figure for CON (**left graphs**) and AIE (**right graphs**). The red box represents the 2-s baseline window for the phasic activity analysis. The thickness of each line reflects the proportion of neurons exhibiting phasic excitation or inhibition (non-phasic cells are not shown). Inset: box plots show the amplitude of excitation during the target window (blue box) for each phasic excitatory cell. OFC phasic activity during PCA baseline is presented for CS onset (**A**), CS offset (**B**), and first receptacle entry after CS offset (**C**). A higher percentage of phasic excitatory cells were recorded during the PCA baseline in AIE-exposed rats. However, no difference was observed in group comparisons on the amplitude of the firing activity displayed by phasic excitatory cells for these events (**A-C**). OFC phasic activity was also analyzed during omission sessions for CS onset (**D**), CS offset (**E**), and first receptacle entry after CS offset (**F**). There was a larger percentage of excitatory cells after CS onset in OFC neurons recorded from AIE-exposed rats. In contrast to the baseline session, both groups presented similar percentages of excitatory cells after CS offset and during the first receptacle entry after CS offset. No AIE effect was observed in the amplitude of the phasic excitation for these events. Neuronal phasic activity data are presented as mean firing rate. Box plots showed the median, interquartile range, and minimum and maximum data values, with CON in gray and AIE in orange. Note that the percentage of neurons displaying phasic activity are descriptive results. For statistical details of the analyses on the amplitude of phasic excitatory cells activity, see Supplemental Table S2.

Initially, OFC neuronal population activity during the baseline session was analyzed by creating peri-stimulus time histograms centered at CS onset, CS offset, and the first receptacle entry after the CS offset when the animal aimed to retrieve the US (see Supplemental Fig. S4 for examples of individual neuron activity). We observed a higher mean firing rate in OFC-recorded neurons of AIE-exposed rats compared to CON-exposed rats (Fig. 3B and C) in response to both CS onset and CS offset (*P* ≤ 0.05; Table 2). In contrast, no AIE effect was observed on the OFC population firing rate during the receptacle entry that followed the CS offset event (*P* > 0.05; Fig. 3D; Table 2). Next, we determined whether individual neurons displayed phasic excitation, phasic inhibition, or neither based on their firing pattern at these events (Fig. 4A,B, and C). No significant group differences were found for the peak firing rates of the phasically excited neurons (P’s > 0.05; see Supplemental Table S2). We observed a higher percentage of neurons classified as excitatory after CS onset in the AIE group (25% than in the CON group (15%), although this difference is descriptive rather than statistical. Thus, the greater excitation in the OFC population recorded in AIE versus CON rats may be due to more neurons exhibiting excitations rather than larger amplitude. Another possibility is that neither number nor amplitude are significant, but they each contribute to the overall effect. However, this finding must be replicated.

**Table 2:** Results of the statistical analysis on the electrophysiological data from Pavlovian conditioned approach baseline and reward omission session on sign-tracking rats.

I. OFC and NAc neuronal population activity (mean firing rate of all recorded neurons) during baseline session								
OFC NAc								
Variable		MWU (U)	*P*			MWU (U)	*P*	
CS onset		1298	**0.05**			729	0.65	
CS offset		1253	**0.02**			647	0.21	
RE after CS offset		1537	0.53			653	0.23	
II. OFC and NAc neuronal population activity (mean firing rate of all recorded neurons) during omission session								
CS onset		1436	**0.04**			680	0.53	
CS offset		1719	0.56			676	0.50	
RE after CS offset		1697	0.49			738	0.96	
III. Mixed-linear model (MLM) analysis comparing the possible influences of OFC neuronal firing rate after CS onset and lever presses 10 before CS offset								
Session Baseline Omission								
	F			*P*	F			*P*
Exposure	9.176			**0.003**	0.002			0.963
LP 10 s before CS onset × firing rate	4.250			**0.042**	0.000			0.991
Interaction	6.062			**0.015**	2.427			0.122
IV. OFC individual neuronal firing rate and subsequent rat behavioral response: correlational slope comparison between control and AIE group								
Session Baseline Omission								
	T-test (t)			*P*	T-test (t)			*P*
CS onset and LP 10 s before CS offset	2.65			**0.009**	0.40			0.68
CS offset and RE 10 s before CS offset	5.07			** < 0.001**	0.21			0.82

Group comparisons on OFC and NAc population neuronal activity were conducted using Mann–Whitney U test. Analyses on OFC population activity across baseline and omission sessions were carried out using generalized linear model with a Poisson distribution and a log link function. Underlined type indicates significant differences (*p* values are described on the table). Bold underlined type indicates significant differences (see methods). CS (conditional stimulus); LP (lever presses); NAc (nucleus accumbens); OFC (orbitofrontal cortex); RE (receptacle entries).

To evaluate whether AIE exposure could alter neuronal activity in response to changes in the CS-US contingency, we recorded neuronal activity in the same rats during the reward omission session. Consistent with the neuronal firing during the baseline session, we observed that the mean firing rate of OFC population activity in response to the CS onset was significantly higher in the AIE group than in the CON group (*P* ≤ 0.05; Fig. 3E; Table 2). This result demonstrates a consistent change in OFC activity in response to the CS onset promoted by AIE, an effect that was observed even in the absence of the US. In contrast, no group difference was observed in OFC population activity in response to the CS offset (*P* > 0.05; Fig. 3F; Table 2) or at the receptacle entry after the CS offset (*P* > 0.05; Fig. 3G; Table 2). No significant group differences were found for the peak firing rates of the phasically excited neurons (P’s > 0.05; Fig. 4D, E, and F; Supplemental Table S2). Additionally, no AIE effects on OFC activity during behavioral responses were observed in either session (*P* > 0.05; Supplemental Fig. S5 and S6; Supplemental Table S2). Therefore, the present study found that AIE exposure induced long-term effects on OFC activity, which preferentially impacted OFC neural activity to the CS presentation rather than during the subsequent conditional behaviors.

### AIE exposure did not alter NAc neuronal activity in response to the CS or conditioned approach

**Figure 5:**
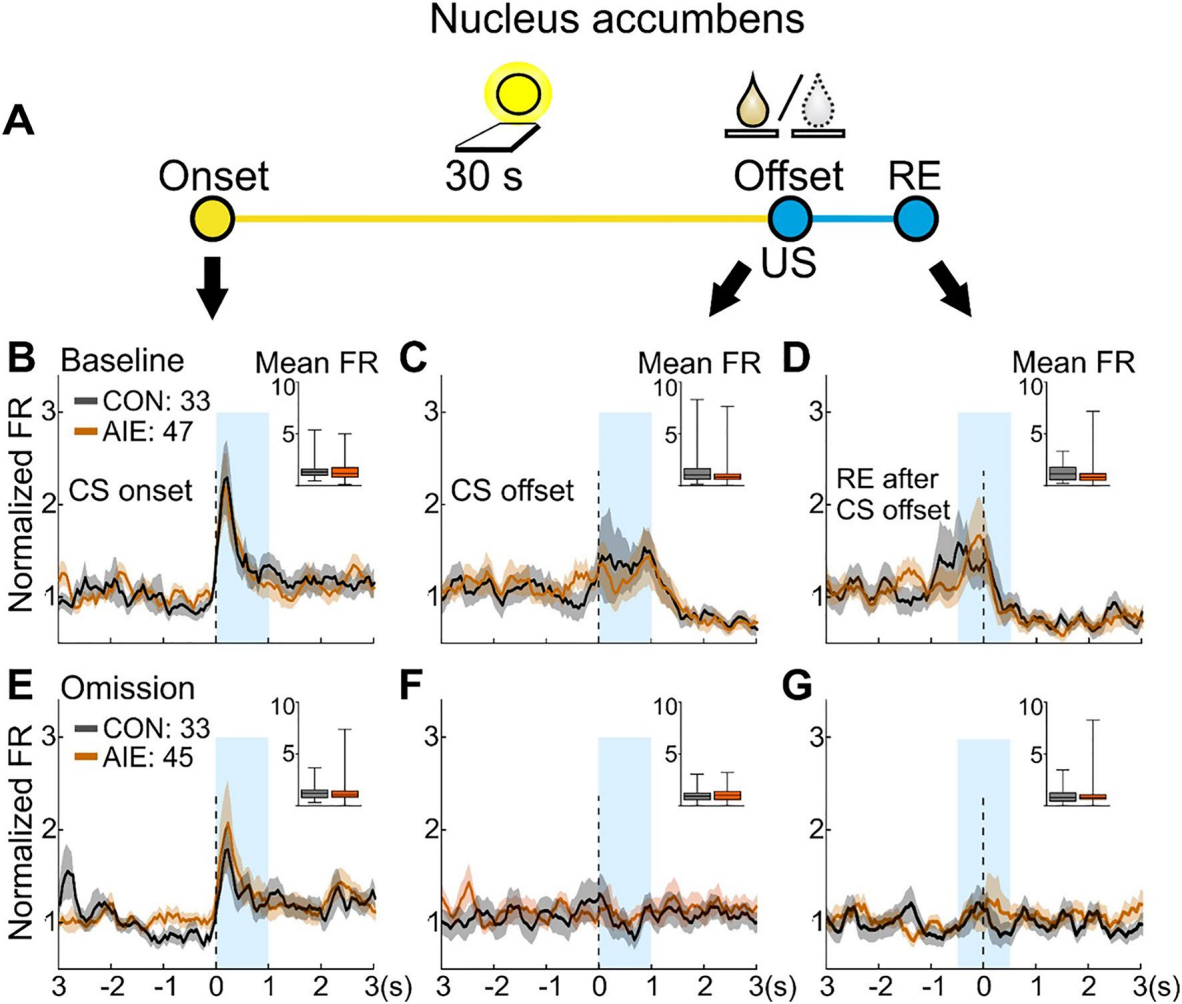
No AIE effect was observed on NAc activity in response to CS onset, CS offset, and receptacle entry after CS offset. Single-unit activity of NAc neurons were analyzed in sign-tracking rats during the baseline and omission sessions. (**A**). Illustrative representation of each trial, as described for Fig. 3. (**B-D**). NAc population activity (average of all neurons) during baseline session. (**B**). NAc neuron population activity did not differ between exposure groups after CS onset during the PCA baseline session. (**C**). NAc neuron population activity did not differ between exposure groups after CS offset. (**D**). NAc neuron population activity did not differ between exposure groups during the first receptacle entry to retrieve the US. (**E–G**). NAc population activity during the omission session. (**E**). After changes in CS-US contingency, NAc neuron population activity did not differ between exposure groups after CS onset. (**F**). NAc neuron population activity did not differ between exposure groups after CS offset. (**G**). NAc neuron population activity did not differ between exposure groups during the first receptacle entry after CS offset. Neuronal population activity data are normalized to the whole session firing rate and presented as mean firing rate (line) ± SEM (shading) of all recorded neurons for CON (gray) and AIE (orange) groups. Number of recorded cells from NAc during baseline session: 33 cells from CON and 47 cells from AIE group. Number of recorded cells from NAc during omission session: 33 cells from CON and 45 cells from AIE group. Inset: box plots show the mean firing rate in the target window (blue box). These data are presented as median, interquartile range, and minimum and maximum data values. For statistical details see Table 2.

**Figure 6:**
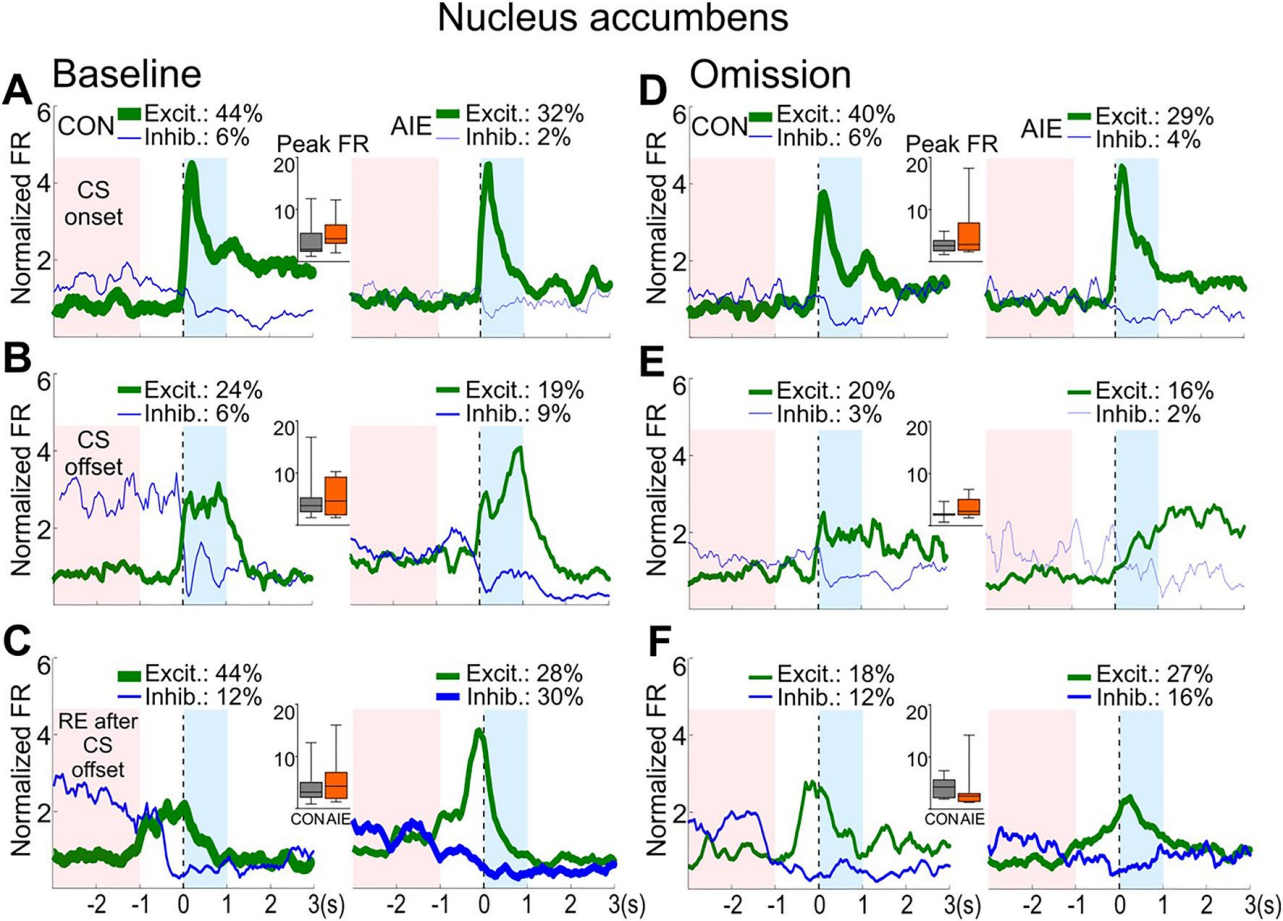
No AIE effect was observed on NAc phasic activity after CS onset, CS offset, or the first receptacle entry after CS offset. Phasic activity in NAc neurons was analyzed during PCA baseline and omission sessions. Firing activity for neurons that showed phasic excitatory activity (in green) and phasic inhibitory activity (in blue) are shown in each figure for CON (**left graphs**) and AIE (**right graphs**). The red box represents the 2-s baseline window for the phasic activity analysis. The thickness of each line reflects the proportion of neurons exhibiting phasic excitation or inhibition (non-phasic cells are not shown). Inset: box plots show the amplitude of excitation during the target window (blue box) for each phasic excitatory cell. NAc phasic activity during the baseline session (**A**, **B,** and **C**) and omission session (**D**, **E,** and **F**) are presented for CS onset, CS offset, and the first receptacle entry (RE) after CS offset, respectively. No AIE effect was observed during these events on the amplitude of the activity displayed by phasic excitatory cells during both recording sessions. Neuronal phasic activity data are presented as mean firing rate. Box plots showed the median, interquartile range, and minimum and maximum data values, with CON in gray and AIE in orange. Note that the percentage of neurons displaying phasic activity are descriptive results. For statistical details of the analyses on the amplitude of phasic excitatory cells activity, see Supplemental Table S2.

We next performed the same analyses on NAc neuronal firing patterns during PCA baseline and reward omission sessions (Fig. 5A). As expected, a robust excitation to the CS onset was observed in NAc neurons in AIE and CON rats, and a lesser excitation to the CS offset and on receptacle entry after CS offset (Fig. 5B–G and Fig. 6; for statistical details see Table 2 and Supplemental Table S2). Nevertheless, no group differences were observed in NAc population activity on phasic activity to CS onset, CS offset or receptacle entry after CS offset in either the baseline or the reward omission sessions (P’s > 0.05).

Analyses on NAc activity during behavioral responses were also performed. The NAc population firing rate presented little change during lever presses and during receptacle entries before CS onset (i.e., unconditioned entries) in both baseline and omission sessions (P’s > 0.05; Supplemental Fig. S7 and S8; Supplemental Table S2). However, NAc neurons recorded in the AIE group fired significantly less at the first receptacle entry during the CS period (i.e., conditioned entry) compared to those recorded from the CON group during the baseline session (*P* < 0.01), but not during the omission session (*P* > 0.05, Supplemental Fig. S7 C and F; Supplemental Table S2).

As we observed robust excitation in OFC and NAc in response to CS onset and CS offset events, we explored whether the OFC and NAc firing responses to these events were correlated within rats. No significant correlations between OFC and NAc neuronal activity for these events were observed during baseline or reward omission sessions (P’s > 0.05; Supplemental Fig. S9). In summary, while cue-evoked phasic excitation was generally greater in the NAc than in the OFC, AIE exposure did not enhance cue-evoked neural activity in this region. In contrast, AIE exposure diminished neuronal phasic firing associated with conditioned receptacle entry.

### Does neural activity in the OFC predict subsequent conditioned approach?

**Figure 7:**
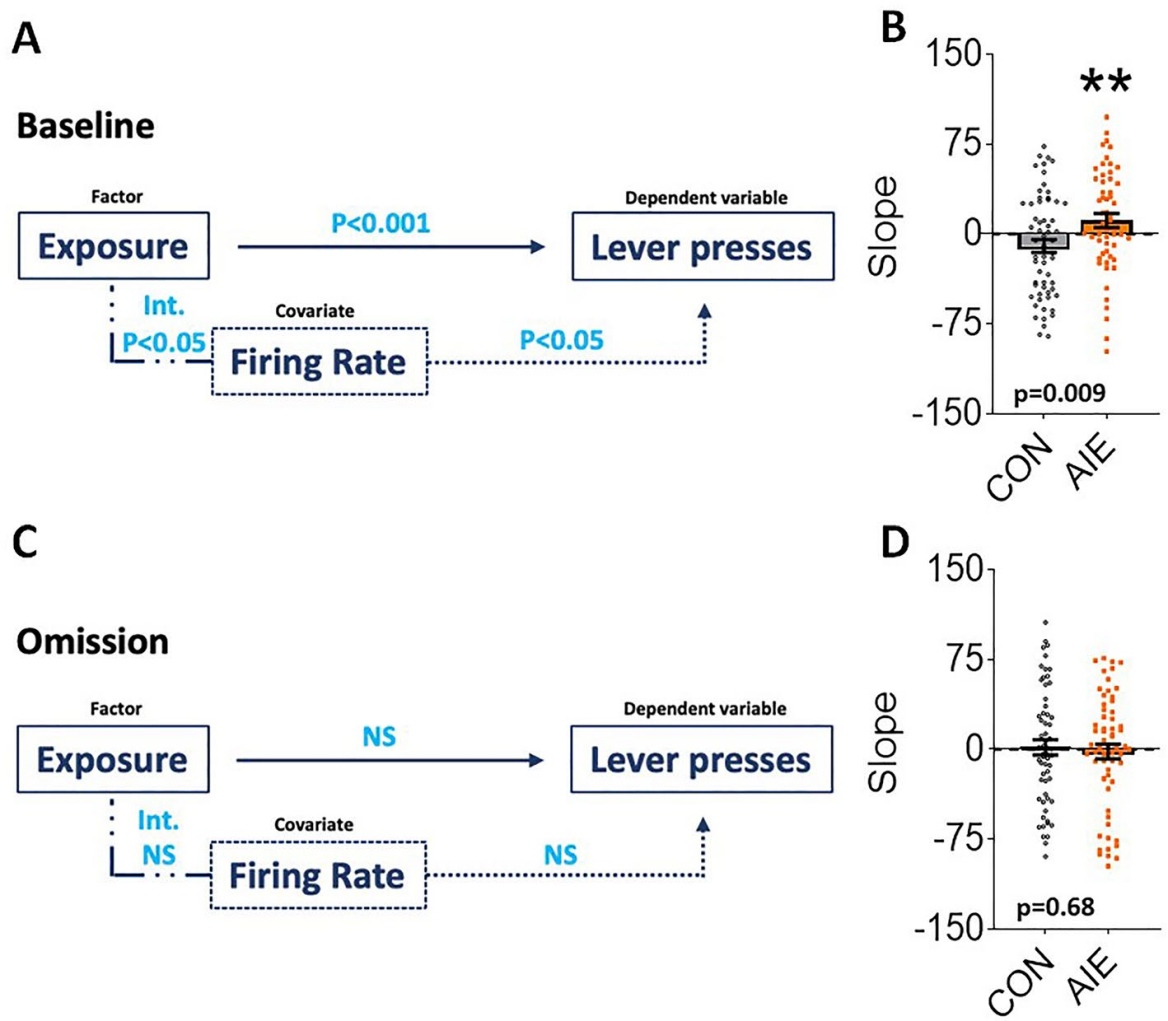
OFC firing response to CS onset is associated with subsequent lever presses in AIE-exposed, but not CON-exposed, rats. The results of the mixed-linear model (MLM) analysis are shown for baseline (**A-B**) and omission sessions (**C-D**). The left panels (**A** and **C**) represent the comparison of exposure (AIE and control), OFC mean firing rate (FR) to CS onset (CS on), and lever presses during the last 10 s before CS offset. The slope values represent the comparison of individual OFC neurons' activity to CS onset and LP 10 s before CS offset. Group comparisons are shown on the right bar graphs (**B** and **D**). Solid lines represent the main effect of exposure on lever presses. Dotted lines represent the main effect of firing rate on lever presses. Intermittent dotted lines represent the exposure × firing rate interaction. **P < 0.01. For statistical details see Table 2 and Supplemental Table S2. Int. (interaction), NS (non-significant).

The start and end of the CS convey distinct information about the forthcoming US, implying that modifications in neuronal firing rates to the CS onset and offset in AIE and CON animals might reflect distinct associations. To be specific, the CS onset signifies the commencement of the trial and anticipates its termination (CS offset), whereas the CS offset is a closer predictor of the sucrose reward (US). During the baseline session, OFC neuronal firing rates to CS onset and CS offset increased in AIE-exposed rats compared to controls. It is known that the time between a cue and a reward influences behavior. The behavior associated with the US tends to occur predominantly towards the end of the interval, reflecting the anticipation of the reward^30,31^. Since our CS lasts for 30 s, we expected an increase in lever presses in proximity to the end of the 30-s interval. As demonstrated in Fig. 2I–L, rats from both groups performed more lever responses during the last 10 s in both sessions. As AIE animals exhibited an increased OFC firing rate at CS onset (Fig. 3), we asked whether the neural activity at CS onset predicted subsequent lever presses in AIE animals. To test this, we performed a linear mixed-effects model (LMM), using lever presses as our dependent variable, groups, and rat ID as factors, and rate (individual neuron firing rate) as a covariate. For the baseline session, we observed main effects of both exposure and firing rate on lever presses 10 s before CS offset (Fig. 7A; both P's < 0.05; Table 2), as well as an interaction between these factors. These results indicated that both exposure and firing rate, and an interaction between the two, impact subsequent lever presses. To better understand the interaction and the directional relationship between neural activity and behavior, we used the slope (m) as a metric to capture both firing rate and neural activity. Specifically, we calculated the slope of firing rate and lever presses 10 s before CS offset for each individual neuron included in the previous analysis, then compared the slope values between the groups. We found a significant difference of exposure (Fig. 7A, right; P = 0.009; Table 2), with neurons from AIE animals showing a positive slope and those from control animals showing a negative slope, indicating opposite directional associations between neural activity and subsequent lever presses. However, this effect is lost during reward omission (Fig. 7B; all P's > 0.05; Table 2). Together, these data suggest an effect of neural activity in the OFC at CS onset on subsequent lever presses at baseline.

We also asked whether the OFC response to the CS offset event and receptacle entries after the CS offset were associated. While LMM analysis during baseline session demonstrated a significant exposure effect (*P* < 0.001), no significant firing rate or interaction effects were observed (all P's > 0.05; Supplemental Table S1). Moreover, no differences were observed during the omission session (all P's > 0.05; Supplemental Table S1).

Thus, these analyses indicate that adolescent alcohol exposure differentially alters neural firing to specific components of the CS, depending on the CS-US contingency. Together, our data suggest a relationship between neural activity in the OFC and the behavioral phenotype associated with an increased bias towards reward-associated cues in AIE animals, but not in controls.

## Discussion

This study examined the long-lasting effects of adolescent alcohol exposure in adult female rats on associative learning (PCA) and neuronal firing activity in the OFC and the NAc. Our results show that rats with a history of alcohol exposure exhibited enhanced excitation in the OFC to a reward-predictive cue compared to controls, despite similar behavioral responses to the cue. Specifically, during the baseline session, AIE-exposed rats showed increased OFC population firing upon both CS onset and CS offset. Moreover, OFC firing at CS onset predicted lever presses leading up to CS offset, but this relationship was only observed in AIE-exposed rats. When the US delivery was withheld, AIE rats continued to exhibit enhanced excitation to CS onset, but that firing rate no longer predicted lever presses and the firing rate to cue offset was no longer different from control rats. While neurons in the NAc exhibited CS-evoked excitations, no differences were observed between exposure groups. Thus, exposure to binge levels of alcohol during adolescence can induce long-lasting changes in neural activity in the OFC, influencing different associative learning processes. Although it is known that AIE affects prefrontal physiology and various behavioral parameters, our most important finding is the differences in how OFC encodes information about CS components regarding time and/or association features and how those changes are impacted by adolescent ethanol in female rats, several weeks after the last alcohol exposure.

Long-lasting AIE effects are well documented in male and female pre-clinical models, including impairments in memory^8^, reversal learning^9,10^, attention^32^, increased risk-taking preference and impulsivity^33^, and increasing ethanol consumption in adulthood^34^. Although these AIE effects are well described in the literature, most of these studies were conducted mainly in male subjects^35^. However, a growing literature reports sex differences in AIE effects, including the description that compared to male, female rats exhibit fewer dopaminergic D1 and serotonergic 5-HT_2A_ receptors in frontal cortex and hippocampus^36^, higher expression of impulsive behavior^37^, greater reliance on habitual response strategy^38^, and no alteration on social anxiety-like behavior after AIE^39^. On the other hand, while we previously reported sex differences in sign-tracking, with females exhibiting more sign-tracking behavior than males, AIE produced main effects to promote sign-tracking and reduce goal-tracking in both males and females^26^. Sign-tracking behavior is associated with impulsive behaviors and addiction vulnerability^24,25^, and Healey et al.^37^ demonstrated that, compared to males, AIE-exposed female rats exhibit increased impulsive behavior. In the present study, female rats expressed high levels of sign-tracking over goal-tracking, independently of exposure. Sign-trackers can express a substantial goal-tracking response^40^, which we also observed in this study. This broader spectrum of behavioral responses can be evoked in PCA protocols using a long duration (30 s) CS^26,41^, in contrast to protocols with shorter a CS duration^40^.

We hypothesized that AIE exposure could promote changes in OFC and NAc neuronal activity during associative learning. This hypothesis was supported by the important role of these two regions during PCA^9,11,14,19,20,28,42^. Our findings demonstrated that AIE exposure increased OFC neuronal firing in response to CS onset and CS offset events during regular PCA. In contrast to OFC, no AIE effect was observed on Nac firing activity in response to these events. The OFC effects we observed are consistent with clinical and pre-clinical studies supporting the idea that multiple drugs of abuse impact OFC activity^28,43–45^, including MRI studies showing that cocaine-associated cues elicit OFC activation^46^. Cocaine administration increased drug-specific action-coding activity on the OFC^47^ and acute nicotine exposure blunted firing activity in response to CS onset^28^. To our knowledge, this is the first study to report AIE effects on OFC neuronal firing activity in female rats. Importantly, these results were observed long after the ethanol exposure, consistent with the persistent nature of many of the effects of binge alcohol exposure during the adolescent period. A previous electrophysiological study using male rats showed that AIE exposure induced changes in the OFC-reward outcome and risk preference relationship, resulting in an increase risk preference and reduced OFC responses to rewards^43^. In our study we did not observe changes in OFC activity during the reward retrieval, but in response to CS onset and offset events. This in part can be explained by the fact that our analyses were focused on sign-tracking animals. The OFC may play a key role in representing an individual’s motivational state, the value associated with expected outcomes^48,49^ and the salience of the CS^49^. The CS acquires motivational salience in sign-tracking individuals^25^, and the enhanced OFC firing activity to a CS may be part of the functional system supporting the elevated sign-tracking response in AIE-exposed animals since OFC activity is described as guiding, rather than to directly producing, the selection and initiation of the encoded actions^50^. This argument is important since OFC is only one among multiple components of a broader circuit implicated in sign-tracking behavior^24,25^. Our study also evaluated the AIE effect on the proportion of phasic excitatory/inhibitory activity of OFC in response to the CS events. However, due to the low number of recorded neurons we were underpowered to properly address this question. We recommend that future studies should more closely evaluate the potential changes in the OFC phasic excitatory and/or inhibitory activity induced by AIE exposure.

Another important function attributed to the OFC is encoding the expected value of an outcome^51^. We performed electrophysiological recordings during reward omission and observed elevated OFC firing after CS onset in AIE-exposed rats, similar to activity in the PCA baseline session. However, OFC firing activity after CS offset in AIE rats decreased to CON levels. Namboodiri et al.^30^ using a CS with short duration (2 s), demonstrated that clusters of OFC neurons encoded different CS-US memory representations. Recordings of OFC activity after multiple extinction sessions demonstrated a stable CS-US representation by OFC activity in specific OFC clusters, which is similar to our findings of OFC activity after CS onset. OFC neural activity in response to CS onset and CS offset may encode different aspects of the task. Under baseline conditions, AIE increased neural activity to CS elements, strengthening the relationship between each event (CS onset and offset) with the US. When the expected outcome was absent during the omission session, OFC activity decreased to CS offset but not CS onset, specifically in AIE-exposed rats, while in the control group activity remained low. Indeed, neuronal activity to CS offset in control-exposed rats was low under baseline conditions, making it difficult to identify changes during the omission session. Nevertheless, at least in AIE-exposed rats, we argue that CS offset, but not CS onset, is functionally linked to the US. Supporting this interpretation, in the absence of an expected US, OFC function would be required to update reward predictions based on the new outcome, guiding the proper update of behavioral responses^52^. Indeed, in the absence of the US, we observed a decrease in the number of receptacle entries across the session in both groups.

As OFC and other cortical regions are still undergoing developmental maturation during adolescence^1,2^, AIE exposure can alter cortical maturation, which may produce long-lasting impairment in decision-making processes^2^, enhance impulsive behaviors^53^ and contribute to behavioral inflexibility^16^. Our results and others^9,43^ demonstrate that AIE exposure could induce maladaptive development of cortical structures resulting in long-term effects on AIE-exposed individuals. Coleman et al.^9^ demonstrated that AIE can lead to cognitive dysfunction by disrupting OFC neuronal maturation processes. The authors demonstrated long-term AIE-induced anatomical changes in OFC due to an increase in levels of immature (juvenile) forms of extracellular matrix proteins. Also, other AIE-induced effects that could be associated with the results observed in our study are the long-term changes in OFC functional connectivity with cortical and limbic structures^11,14^, leading to deficits in behavioral flexibility. Therefore, AIE effects on OFC-firing in response to CS observed in our study may reflect a broader impairment in neurodevelopment induced by AIE exposure. A limitation of our study is that only females were examined. While we previously^26^ did not observe AIE sex-dependent effects in sign-tracking behavior, more studies are required to confirm if the AIE effects in the OFC response to CS onset observed in our study could also be observed in male subjects.

We hypothesized that AIE exposure would induce changes in NAc response to reward-associated stimuli. AIE exposure has been shown to induce changes in NAc activity, including changes in dopamine release^13,54^ and an increase intrinsic excitability in medium spiny neurons^19^. Contrary to our hypothesis, no AIE-induced changes were observed on NAc firing activity in response to CS onset and CS offset events. Consistent with previous studies^18,42^, we observed that NAc neurons exhibit robust neuronal activity in response to CS onset, which is more pronounced compared to CS offset. Also, after changes in CS-US contingency, NAc-recorded neurons in both CON and AIE-exposed groups still show robust firing activity in response to CS onset, similar to previous reports in sign-tracking rats^42^. We also questioned whether AIE induced changes in NAc firing occurs during conditioned responses. We demonstrated that neurons recorded in CON-exposed rats exhibit substantial excitatory activity during cue-evoked reward-receptacle approach. However, significantly less NAc excitation was observed in the neurons recorded in the AIE-exposed group. As NAc activity is required during PCA and approach behavior^55,56^, this finding suggests an AIE-exposure effect on NAc encoding of conditioned approach. A caveat is that the modest number of neurons recorded from NAc is a limitation to this study that tempers our populational and phasic activity analysis and conclusions regarding AIE effects on NAc neuronal activity. Future studies should more closely address AIE’s effects on NAc encoding of reward-associated cues, conditioned approach, and potential NAc-OFC interaction during PCA to provide a better understanding of possible AIE effects on NAc.

In summary, we demonstrated that AIE exposure induces long-lasting effects on OFC neural responses to reward-associated stimuli. Our results showed that AIE-exposure increases OFC firing rate in response to CS onset and CS offset events in sign-tracking female rats. Even in the absence of the US delivery, AIE-exposed rats exhibited increased OFC activity to CS onset compared to control-exposed rats. Also, we showed that in AIE-exposed, but not control-exposed rats, the OFC firing rate in response to CS onset predicts subsequent lever press behavior. We propose that this AIE-induced enhancement in OFC neural activity associated with these CS events could be associated with a higher cue sensitivity, being potentially involved in the AIE behavioral deficits observed in other studies^10,12,26,43^, although additional studies are required to confirm it. Our findings highlight the importance of CS features and suggest a new line of research about AIE, brain development and reward-associated cues as predictors of biased and inflexible behavior.

## Methods

### Animals

Female Sprague–Dawley rats (n = 40) bred in-house were pair-housed with a sibling during the alcohol exposure and initial training period. Animals received food and water ad libitum during the entire study and were housed in a temperature- and humidity-controlled vivarium with a 12:12 h light–dark cycle (lights on at 07:00). All experiments occurred during the light cycle. Experimental procedures were performed in accordance with the NIH Guide for Care and Use of Laboratory Animals and approved by the Institutional Animal Care and Use Committee of the University of North Carolina at Chapel Hill. The study is reported in accordance with ARRIVE guidelines (https://arriveguidelines.org).

### Adolescent alcohol exposure

Alcohol exposure and behavioral training procedures were performed as previously described^26^. Starting at postnatal day (P) 25, adolescent intermittent ethanol (AIE) or control exposure began. Rats received 5 g/kg of intragastric ethanol (25% v/v in water) or the equivalent volume of water (CON). The administration was performed once per day on a 2-days-on, 2-days-off regimen through P54, completing a total of 14 doses. We previously reported that this regimen did not affect developmental weight gain in females and produced blood ethanol concentrations of approximately 230 mg/dl at 60 min post-administration^26^.

### Pavlovian conditioning

Between P68 and P70, rats started PCA training. One hour prior to the beginning of the first session, a bottle of 20% sucrose (w/v in water) was placed in the rats’ home cages, allowing them to familiarize themselves with the US solution. Next, the animals were placed in standard behavior chambers (MedAssociates, St. Albans, VT). Each of the behavior chambers contained a receptacle for dispensing liquid rewards, a photobeam detector to record receptacle entries, a cue light, and a retractable lever positioned below the cue light. White noise and a house light were active throughout the session. During the first training session, rats became familiar with non-contingent US delivery at the receptacle. In this initial session, 15 US deliveries (0.1 ml of 20% sucrose) were made on a variable inter-trial interval of 60–300 s (s), and no CS was presented. Subsequent PCA sessions occurred daily on a Monday—Saturday schedule. Animals were placed in the behavioral chambers 5-min prior to the start of the session. During each PCA session, 15 CS-US trials occurred with a variable inter-trial interval of 60 to 300 s. Each trial consisted of a 30 s presentation of the CS (cue light illumination concurrent with the lever extension). The CS offset (cue light off and lever retraction) was immediately followed by the delivery of the US at the receptacle. After 15 training sessions, animals were separated into individual housing cages and 5 additional sessions were performed. Next, rats underwent 5 PCA sessions in larger, custom-built Plexiglas chambers (MedAssociates) that were similar to the training chamber, but optimized for electrophysiology recording, with angled walls to prevent the electrophysiological headstage from hitting the walls of the chamber.

### Surgery

After the habituation to the custom-built chambers, stereotaxic surgery was performed for electrode array implantation. Rats were anesthetized with isoflurane (5% induction, 2–3% maintenance). During the surgery, two microwire electrode arrays were implanted; each array contained 8 stainless-steel Teflon-coated wires, 50-μm in diameter, and spaced 0.5 mm apart with a 2 × 4 configuration (NB Labs, Denison, TX). One array was placed in the OFC (3.7 mm anterior, 2.6 mm lateral from bregma, 5.0 mm ventral from the adjacent skull surface) and the second array was placed in the contralateral NAc, (targeting the core subregion, 1.7 mm anterior, 1.5 mm lateral from bregma and 7.4 mm ventral from the adjacent skull surface), with side counterbalanced across animals. After the surgery, animals were monitored and received 50 mg/kg meloxicam s.c. daily for 3 days.

### Behavioral and electrophysiology experiments

After at least ~ 7 days of recovery from surgery, PCA sessions resumed in the customized behavior chambers. The animals first underwent 1–2 PCA sessions to reinstate the conditioned behavior. During the next 4 sessions, the animals were gradually habituated to a flexible tether that connected electrode arrays to the headstage assembly. Thereafter, rats were tethered in all sessions.

Electrophysiological data was recorded during two sessions: a PCA baseline (typical) session followed by a reward omission session. The reward omission session was performed in order to evaluate behavioral flexibility due to changes in US availability^57^. During the omission session, no US was delivered after any of the 15 CS presentations. Specifically, during this session, the US delivery system was still filled with the sucrose solution, but it was not connected to the delivery pump. Therefore, the rats could still smell the sucrose solution and the CS offset event still activated the delivery pump, but no US was delivered. Importantly, the delivery pump was placed outside of the behavioral chambers, therefore avoiding a potential influence of the delivery pump activation sound on behavioral responses and on neuronal firing activity.

During the PCA baseline and omission sessions, neuronal activity was recorded as previously described^28,58,59^ using a multichannel acquisition processor (MAP system with SortClient software; Plexon Inc., Dallas, TX, USA). For the recording sessions, the animals were tethered and placed in the behavioral chamber 15 min before the start of the PCA session. During this time the threshold setting for the electrode channels was set, and one of the channels in each array was manually selected as a differential reference channel for all channels in that array. During PCA sessions, MedAssociates software provided timestamps of the behavioral events (CS onset, lever presses, US delivery, and receptacle entry) that were temporally aligned by the MAP system with the electrophysiological recordings.

### Histology

Animals were anesthetized using 1.5 g/kg urethane i.p. (50% w/w in saline). A 10-μA current was applied for approximately 5 s to each stainless-steel wire to produce an iron deposit at the electrode tip, allowing determination of the recording site. Next, rats were perfused with a formaldehyde solution and the brains were removed. Histological confirmation of electrode placement was performed as previously described^28^. Briefly, 40-μm brain slices were taken on a cryostat. The slices were stained with potassium ferracyanide and thionin to determine the electrode placements and compared to a rat brain atlas to map the OFC and NAc electrode placements. Only data from microwires confirmed to reside in the target regions were included in the analysis (Supplemental Fig. S2).

### Data analyses

#### Behavior data analyses

Behavioral data were digitally collected by MedAssociates software during all PCA sessions. The following behavioral metrics were analyzed: receptacle entries (30 s before CS onset, during CS onset and 10 s after CS offset), receptacle elevation score, lever presses, latencies to press the lever and to approach the reward receptacle (after CS onset and after offset to retrieve the US), and probabilities of receptacle entry and lever press during CS. The receptacle elevation score was calculated as the number of receptacle entries during the CS presentation minus the number of entries during the 30 s prior to the CS presentation; this metric reveals the conditioned response promoted by CS^60^. Probabilities were calculated based on the number of trials when the animal performed the specific behavior (lever press or receptacle entry) at least once divided by 15 (total number of trials). As our PCA protocol uses a long CS time (30 s), in order to better understand the sign and goal-tracking responses surrounding the CS onset/offset events we accounted for the number of lever presses and receptacle entries performed 10 s after CS onset, 10 s before CS offset as well as receptacle entries performed 10 s after CS offset. To describe the relative performance of sign-tracking and goal-tracking conditioned responses, we adapted a ST-GT formula previously described by Madayag et al.^26^ that included number of lever presses, elevation score, response latencies, and response probabilities, as presented in the following formula:

$$\left( {\frac{{{\text{lev}}.{\text{ press}}. - {\text{elev}}.{\text{ score}}}}{{{\text{lev}}.{\text{press}} + {\text{abs}}.{\text{ value of elev}}.{\text{ score}}}}} \right) + \left( {\frac{recept latency - lev. latency}{{30}}} \right) + \left( {\frac{Lev. prob. - Recept. prob.}{{Lev. prob. + Recept.prob.}}} \right) /3$$

The resulting score could range from -1 to + 1, and an animal was classified as “sign-tracking” with a score between + 0.3 to + 1, “intermediate” with a score from + 0.29 to -0.29, and “goal-tracking” with a score between -0.3 to -1. The behavioral data from PCA baseline session was used in the ST-GT score calculation.

#### Neuronal firing analyses

Neuronal activity recorded from individual neurons during baseline and US-omission sessions was sorted using Plexon Offline Sorter software. Units were identified using automated cluster sorting based on principal component analysis and template sorting, informed by signal-to-noise ratios collected during the recording session. Criteria of inclusion included a signal-to-noise ratio ≥ 2 and the existence of specific clusters after the principal component analysis for the sorting process. Timestamped data were imported into NeuroExplorer software (NEX Technologies, Madison AL), for the generation of perievent histograms of firing rates. Custom-written MATLAB programs (Mathworks, Natick, MA) were used to analyze the firing patterns of neurons surrounding experimental events, as previously described^28,58^. Firing rates surrounding behavioral or experimental events were normalized by dividing the firing rate by the mean of the whole session firing rate. For population activity analysis in the NAc and OFC, the normalized neuronal activity was aligned to the events of interest and smoothed with a moving average of 250 ms in 50 ms steps. Neuronal population activity analysis was performed by calculating the mean firing rate of all neurons during selected events. To capture the neuronal firing pattern in response to an external event (CS onset, CS offset), the analysis window was 1 s immediately after the events. Also, we observed that this analysis window for the CS offset would be less affected by the first receptacle entry after the CS offset (reward consumption; Supplemental Fig. S10). To capture the neuronal firing pattern surrounding a conditioned response (lever press, receptacle entry), the analysis window was 500 ms before and 500 ms after the event. To compare neuronal firing between groups, we calculated the mean firing rate during the above analysis windows.

For individual neuronal analysis, a z-score calculation was performed to identify phasic changes in firing rate surrounding a particular event. The z-score calculation considered the mean firing rate during the target window minus the mean firing rate during the baseline window, divided by the standard deviation of firing during the baseline window (2 s prior to the target window). Neurons with a z-score > 2 or < -2 were classified as “excitatory” or “inhibitory” phasic neurons, respectively, and other neurons (z-score between -2 and 2) were classified as “non-phasic” neurons. Firing rate amplitude of excitatory phasic neurons was further analyzed by selection of the peak firing rate during the target window.

Analysis on neuronal population activity and individual neuronal activity were conducted during PCA baseline and reward omission session using different approaches. During PCA baseline, initial analyses during PCA baseline were conducted using all 15 trials. However, during reward omission, initial analyses were performed considering trials 6 to 15, when the animals experienced that no US was available after CS offset.

### Statistical analysis

Behavioral data were assessed using the D’Agostino & Person normality test, and behavioral parameters that did not present a normal distribution were analyzed using nonparametric tests. Behavioral data from the last 5 days of the PCA training phase were analyzed using Mann–Whitney U (MWU) or *t*-test, depending on the normality distribution. Group comparison on the ST-GT score was performed using MWU test. Behavioral data from the PCA baseline and reward omission session were analyzed using a generalized linear regression (GLM) model with a Poisson distribution, a link-log function, and Wald chi-square contrasts. However, for the variables that presented a normal distribution, a repeated-measures 2-way ANOVA was performed.

For the electrophysiological data analysis, comparisons of neuronal population activity and the peak firing rate of excitatory phasic neurons for each group were performed using the MWU test. The GLM model was applied on behavioral and electrophysiological data analysis across the recording sessions. A mixed-linear model (MLM) was used for analysis on possible influences of OFC neuronal firing rate on behavioral responses. For this analysis we used lever presses or receptacle entries as our dependent variables, groups and rat ID as factors, and rat (OFC firing rate 1 s after CS onset and offset events) as our random effect. In addition to the MLM analysis, the linear regression slope values for individual OFC neuron activity (after CS onset or CS offset) and conditioned responses (lever presses 10 s before CS offset and receptacle entries 10 s after CS offset) were calculated using the following formula: Y = mX + b (Y = dependent variable; X = independent variable; m = slope; b = estimated intercept). Correlation analyses on the OFC and NAc activity were conducted using Spearman’s correlation test. For these analyses, the mean firing activity of neurons within a brain region (OFC or NAc) in a rat were averaged during the one second after the CS onset or CS offset events. Only rats with neurons recorded in both OFC and NAc were included in these analyses. Analyses using the GLM and MLM model were performed using SPSS Statistics (IBM, Armonk, New York). The remaining analyses were performed using GraphPad Prism software (San Diego, CA). Statistical differences were considered when *P* ≤ 0.05. Marginal differences were reported when *P* > 0.05 and < 0.1.

### Ethics statement

All animal procedures performed during this study were reviewed and approved by the University of North Carolina at Chapel Hill Institutional Animal Care and Use Committee.

### Supplementary Information

Supplementary Information.

## Data Availability

The raw data supporting the conclusions of this article will be made available by the authors upon request, without undue reservation.
