# Supplementary material for: Adolescent alcohol exposure persistently alters orbitofrontal cortical encoding of Pavlovian conditional stimulus components in female rats
Source: Sci Rep. 2024 Jun 14;14:13775. doi: 10.1038/s41598-024-64036-1 (PMC11178901; doi:10.1038/s41598-024-64036-1)
Supplement: Supplementary file 1 — Supplementary Information. [file 41598_2024_64036_MOESM1_ESM.pdf]

Adolescent alcohol exposure persistently alters orbitofrontal cortical encoding of Pavlovian conditional stimulus components in female rats

Jose A. Pochapski[1†], Alexander Gómez-A[3†], Sierra J. Stringfield[4], Hannah Jaggers[3], Charlotte A. Boettiger[3,5], Claudio Da Cunha[1,2], Donita L. Robinson[3,6*]

[1] Laboratorio de Fisiologia e Farmacologia do Sistema Nervoso Central, Department of Pharmacology, Universidade Federal do Parana, Curitiba, PR, Brazil.

[2] Department of Biochemistry, Universidade Federal do Parana, Curitiba, PR, Brazil.

[3] Bowles Center for Alcohol Studies, University of North Carolina at Chapel Hill, Chapel Hill, NC, United States.

[4] Department of Psychiatry, University of Pittsburgh, Pittsburgh, PA, United States.

[5] Department of Psychology & Neuroscience, University of North Carolina at Chapel Hill, Chapel Hill, NC, United States.

[6] Department of Psychiatry, University of North Carolina at Chapel Hill, Chapel Hill, NC, United States.

† These authors have contributed equally to this work and share first authorship

* Corresponding author, DLR@unc.edu.

Supplemental materials:

- Supplemental results
- Supplemental Tables S1 – S2
- Supplemental Figures S1 – S10

Supplemental Results

2.1. Single-unit recording in OFC during PCA baseline and reward-omission sessions

We determined whether individual OFC neurons displayed phasic excitation, phasic inhibition, or neither based on their firing pattern in response to CS onset, CS offset and first receptacle after CS offset events during the baseline session (Fig. 4 A, B, and C). Both groups presented a more robust excitatory than inhibitory phasic activity. Moreover, a higher proportion of OFC neurons in AIE rats exhibited phasic excitation to the CS onset, offset, and during the receptacle entry as compared to CON rats (AIE: 25%, 31% 50%; CON: 15%, 18%, 36%, respectively). However, no significant group differences were found for the peak firing rates of these phasically excited neurons (P's>0.05; see Supplemental Table S2). When considering phasic firing patterns of these OFC-recorded neurons during the omission session, 35% of neurons from the AIE group exhibited phasic excitation after CS onset, compared to 18% of neurons from the CON group (Fig. 4 D). However, the amplitude of the excitatory activity to CS onset did not differ between groups (P>0.05; Supplemental Table S2). No group differences in phasic activity were observed at CS offset and during the receptacle entry that followed this event (P's>0.05; Supplemental Fig. S5 E and F; Supplemental Table S2).

To explore the relationship between AIE-induced changes in neuronal activity and behavioral responses, the neuronal activity surrounding unconditioned receptacle entries (performed in the 30 s before CS onset) and conditioned responses (first lever press and first receptacle entry after CS onset) in each trial were assessed. No significant differences in firing rate to behavioral responses were observed in the OFC at the population level between AIE and CON groups. Also, individual neurons activity demonstrated similar patterns of phasic excitation in both sessions and no group differences were observed (Supplemental Fig. S5 A to F; Supplemental Fig. S6 A to G; Supplemental Table S2; P's>0.05).

2.2. Single-unit recording in NAc during PCA baseline and reward-omission sessions

We classified individual NAc neuronal activity by phasic firing patterns. A high percentage of neurons in both AIE and CON rats exhibited phasic activity for CS onset, CS offset and receptacle entries after CS offset events (Fig. 6 A to F). However, no group difference was observed either in the proportion of cells showing excitation or in the amplitude of this excitatory activity (all P's>0.05; Supplemental Table S2).

We next analyzed NAc activity during behavioral responses. The NAc population firing rate presented little change during lever presses in both baseline and omission sessions (P's>0.05; Supplemental Fig. S7 and Fig. S8; Supplemental Table S2). However, during PCA baseline, but not during the reward omission session, NAc-recorded neurons from CON rats exhibited a greater mean firing activity at conditioned receptacle entry as compared to AIE rats (P<0.01; Supplemental Fig. S7 C and F; Supplemental Table S2). Underlying this population effect, phasic activity analysis demonstrated that 56% of NAc neurons displayed phasic excitation during the first conditioned receptacle entry in CON rats compared to 6% in AIE rats during the baseline session (Supplemental Fig. S8 C). No additional group differences were observed on populational or phasic activity during receptacle entries before CS onset (i.e., unconditioned entries) in either the baseline or omission sessions (P's>0.05; Supplemental Table S2; Supplemental Fig. S7and Fig. S8).

Supplemental Table S1. Statistical analysis results from PCA baseline and reward omission session on sign-tracking animals.

I. Group comparison on ST-GT score calculated from PCA baseline session data

Variable	MWU (U)	P
ST-GT score	152	0.21

II. Behavioral parameters during PCA baseline and reward omission session

Variable	Effect	Exp(B)	P	Confidence interval Lower	Upper
RE 10 s after CS offset	Exposure	0.872	0.138	0.586	1.300
	Session	0.804	0.352	0.547	1.180
	Exp*Session	2.041	**0.016**	1.141	3.653
RE during CS	Exposure	0.934	0.675	0.494	1.764
	Time	1,211	0.417	0.691	2.124
	Exp*Time	0.961	0.925	0.420	2.200
RE 10s after CS offset per trial across baseline session	Exposure	1.071	**<0.001**	0.547	2.094
	Time	1.625	**<0.001**	0.791	3.339
	Exp*Time	1.477	0.771	0.610	3.577
RE latency after CS offset across baseline	Exposure	1.400	0.155	0.951	2.073
	Time	0.431	**<0.001**	0.311	0.593
	Exp*Time	0.708	0.140	0.447	1.120

Variable	Effect	F	P	Df	
RE latency after CS onset	Exposure	0.928	0.346	1	—
	Session	11.36	**0.003**		
	Exp*Session	0.059	0.809		
RE 30 s before CS onset	Exposure	0.972	0.335	1	—
	Time	2.146	0.158		
	Exp*Time	1.668	0.211		
RE 10 s after CS offset per trial across omission session	Exposure	0.428	0.520	14	—
	Time	9.348	**<0.001**		
	Exp*Time	0.845	0.618		
Lever latency across baseline	Exposure	0.066	0.79	2	
	Time	2.854	***0.06***		
	Exp*Time	3.417	**0.04**		
Lever latency across omission	Exposure	0.004	0.546	2	
	Time	10.28	**<0.001**		
	Exp*Time	0.974	0.198		
RE latency after CS offset across omission	Exposure	1.896	0.183	2	
	Time	20.03	**<0.001**		
	Exp*Time	0.188	0.828		
LP 10 s after CS onset and 10s before CS offset on omission session	Exposure	0.014	0.906	1	
	Time	8.221	**0.009**		
	Exp*Time	0.073	0.788		

Variable	Session	MWU(U)	P		
RE 10s after CS offset	Baseline	21.50	**0.016**	—	—
RE 10s after CS offset	Omission	52.50	0.828	—	—

III. Linear mixed-effect model (LMM) analysis comparing the possible influences of OFC neuronal firing rate after CS offset and receptacle entries 10s after CS offset.

Session	Baseline		Omission	
	F	P	F	P
Exposure	25.377	**<0.001**	0.148	0.701
RE 10s after CS offset x firing rate	1.613	0.207	0.420	0.518
Interaction	0.282	0.597	0.007	0.934

The ST-GT score group comparison was performed using Mann-Whitney U test. Behavioral analyses during baseline and omission session were conducted using a generalized linear model with a Poisson distribution and a log link function or using a two-way ANOVA depending on the data distribution. Group comparison on receptacle entries 10 s after CS offset were performed using Mann-Whitney U test. Blue type indicates significant differences (P values are described on the table). Bold type indicates marginal differences (see methods). ST (sign-tracking), GT (goal-tracking), RE (receptacle entry).

Supplemental Table S2. Data description and statistical analysis results for OFC and NAc basal and whole-session firing rate, and peak of firing activity and percentage of phasic excitatory cells during baseline and omission.

**I. Basal mean firing rate and whole-session firing rate during the baseline session.**				
	**OFC**		**NAc**	
**Variable**	**FR (Hz)**	**SEM (±)**	**FR (Hz)**	**SEM (±)**
CON basal mean FR	3.03	0.41	3.15	0.54
CON whole session FR	3.19	0.36	3.26	0.41
AIE basal mean FR	4.02	0.54	2.81	0.45
AIE whole session FR	4.05	0.41	3.43	0.44
**II. Basal mean firing rate and whole session firing rate during the omission session.**				
CON basal mean FR	4.06	0.58	3.54	0.63
CON whole session FR	4.03	0.43	3.40	0.47
AIE basal mean FR	4.24	0.46	4.19	0.54
AIE whole session FR	3.60	0.35	3.99	0.41
**III. Statistical analysis results on basal and whole session firing rate during the baseline session.**				
	**OFC**		**NAc**	
**Variable**	**MWU (U)**	**P**	**MWU (U)**	**P**
Basal mean FR	857	***0.08***	310	0.47
Whole session firing rate	1348	***0.09***	752	0.82
**IV. Statistical analysis results on basal and whole session firing rate during the omission session.**				
Basal mean FR	1155	0.76	434	0.33
Whole session firing rate	1749	0.67	648	0.34
**V. OFC and NAc neuronal population activity (mean FR) during baseline session.**				
First lever press	1566	0.65	626	0.14
First RE after CS onset	977	0.35	223	**<0.01**
RE before CS onset	1585	0.73	766	0.93
**VI. OFC and NAc neuronal population activity (mean FR) during omission session.**				
First lever press	1598	0.23	574	0.77
RE before CS onset	1693	0.48	713	***0.08***
**VII. Peak of firing activity of phasically excited cells recorded during the baseline session.**				
CS onset	58	>0.99	68	0.11
CS offset	73	0.35	33	0.81
First lever press	51	0.94	31	0.67
First RE after CS onset	35	0.44	12	0.81
RE before CS onset	121	0.30	29	0.35
RE after CS offset	260	0.46	95	0.92
**VIII. Peak of firing activity of phasically excited cells recorded during the omission session.**				
CS onset	108	0.78	61	0.37
CS offset	43	0.28	7	0.71
First lever press	19	0.21	13	0.29
RE before CS onset	120	0.65	6	**0.02**
RE after CS offset	274	0.97	18	0.10

Basal firing activity was calculated during 60 s before the start signal (chamber light onset) for baseline and omission session. Neurons that increased firing activity (z-score < 2 the pre-event z-score) were defined as phasic excitatory cells (see methods). Group comparisons were performed using Mann-Whitney U test. Blue type indicates significant statistical differences. Bold type indicates marginal differences. FR (firing rate), RE (receptacle entry), CS (conditioned stimuli).

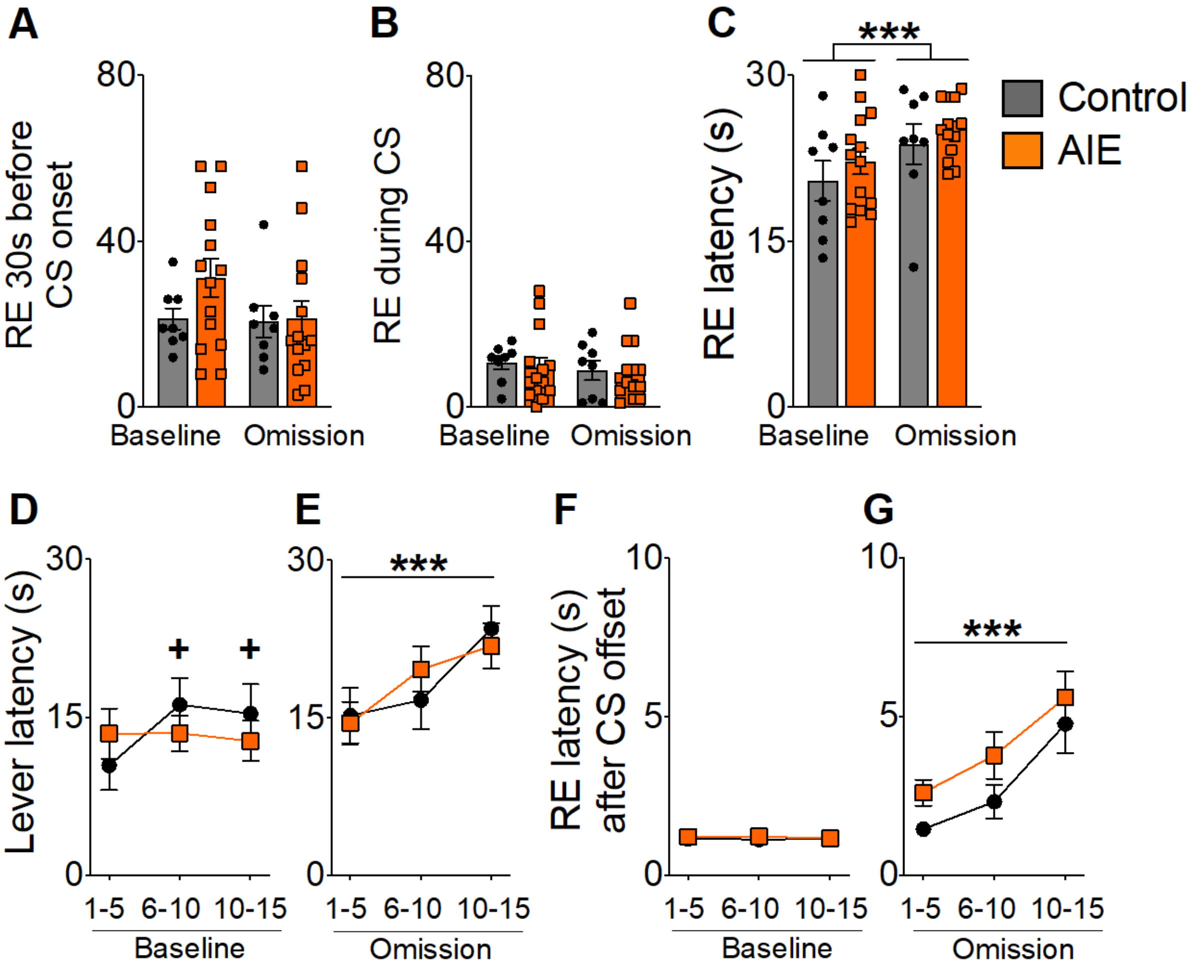

Supplemental Figure S1. AIE exposure did not alter the number of unconditioned receptacle entries, the number of conditioned receptacle entries, or the latency for conditioned receptacle entries. The total number of receptacle entries performed 30 s before CS onset (unconditioned entries), during the 30-s CS period (conditioned entries), and 10 s after CS offset (conditioned entries) were analyzed during baseline and omission session. **A.** No effect of AIE, session, or interaction between factors was observed for the receptacle entries performed 30 s before CS onset. **B.** No statistical difference was observed for receptacle entries during the CS period. **C.** Analysis on latency for the first receptacle entry during CS demonstrated that both groups increased the latency to perform receptacle entries during the omission session, and no effect of AIE or an interaction between factors was observed. **D.** The latency to perform the first lever press was also analyzed across the baseline session. Analysis results demonstrated a significant interaction and also a marginal time effect. However, no treatment effect was observed. Sidak's post-hoc comparison indicated that CON group progressively increased the latency to perform a lever press compared to the initial 5 trials. **E.** The latency to the first lever press analyzed across omission session demonstrated that both groups increased the latency across the omission session. **F.** Further analysis on the receptacle entry latency after CS offset across baseline session demonstrated no significant main effect of exposure or session, neither an interaction between factors. **G.** However, an increase on the receptacle entry latency after CS offset was observed across omission session. However, no exposure or interaction between the factors was observed. Data are expressed as mean ± SEM. The symbols in black for CON (n=8) and orange on AIE (n=14) represent individual subjects' data. *** Main effect of session ($P<0.001$). + $P \leq 0.05$ compared to the first block of trials after Sidak's post-hoc comparison. For statistical details see Supplemental Table S1.

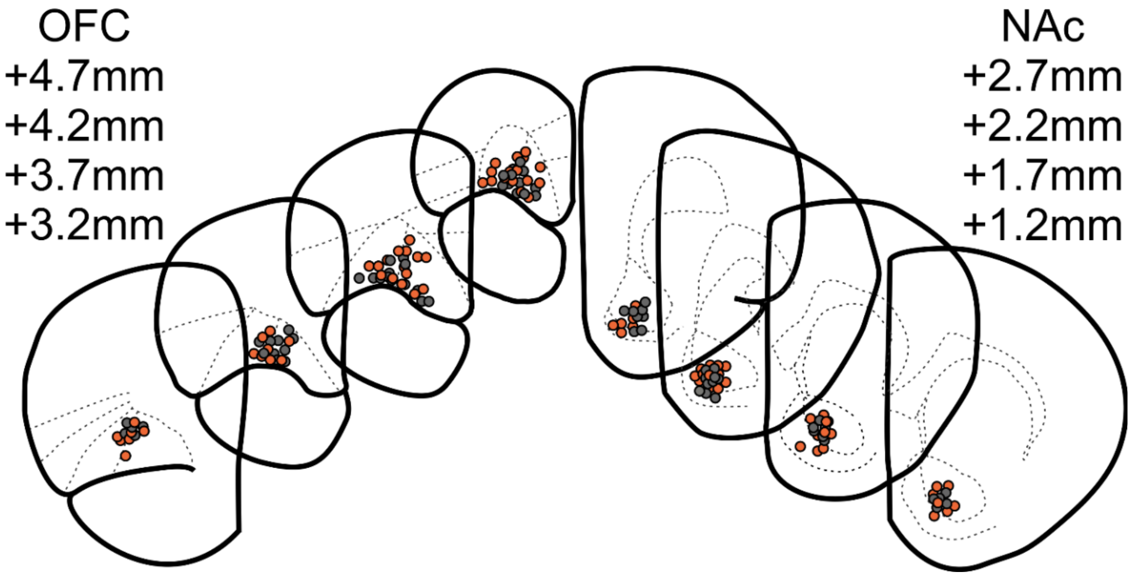

Supplemental Figure S2. Representative illustration of the electrode placements on OFC or NAc. Electrode placements in CON (gray) and AIE group (orange) illustrate the approximated anatomical position of electrode wires in which successful electrophysiological recordings were performed. Anatomic coordinates were obtained from Paxinos and Watson (1998) rat brain atlas, demonstrating the antero-posterior coordinates of the electrode placements on OFC (left) and NAc (right).

Paxinos, G., and C. Watson (1998). "The rat brain in stereotaxic coordinates." San Diego, Academic Press.

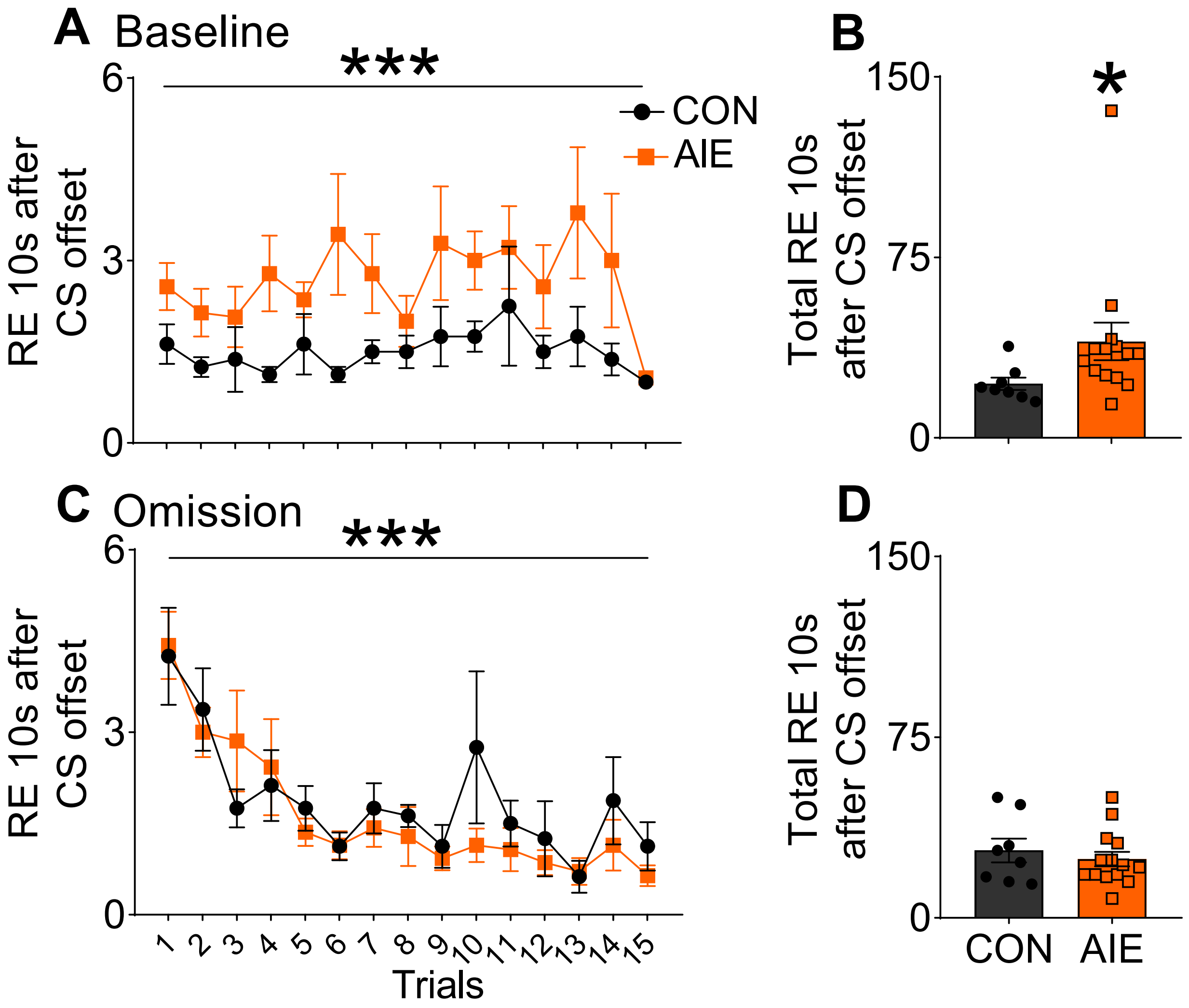

Supplemental Figure S3. Both AIE and CON-exposed groups decreased the number of receptacle entries 10 seconds after CS offset during omission session. A. Receptacle entries performed within 10 s after the CS offset for each of the 15 trials during the baseline session. Significant effects of exposure and time emerged but no interaction between factors. **B.** The total number of entries was collapsed across all 15 trials during the baseline session, and AIE-exposed rats presented a higher number of entries 10 s after CS offset. **C.** Receptacle entries performed within 10 s after the CS offset for each of the 15 trials during the omission session. Both groups significantly decreased the number of entries across the omission session. However, no effect of exposure or interaction between factors was observed. **D.** There was no group difference after collapsing the data across all 15 trials during the omission session. Data are expressed as mean ± SEM. Individual animal data are presented by the symbols in black for CON (n=8) and orange for AIE (n=14). *** Significant main effect of session ($P<0.001$). * Significant group difference ($P\leq 0.05$). For statistical details see Supplemental Table S1.

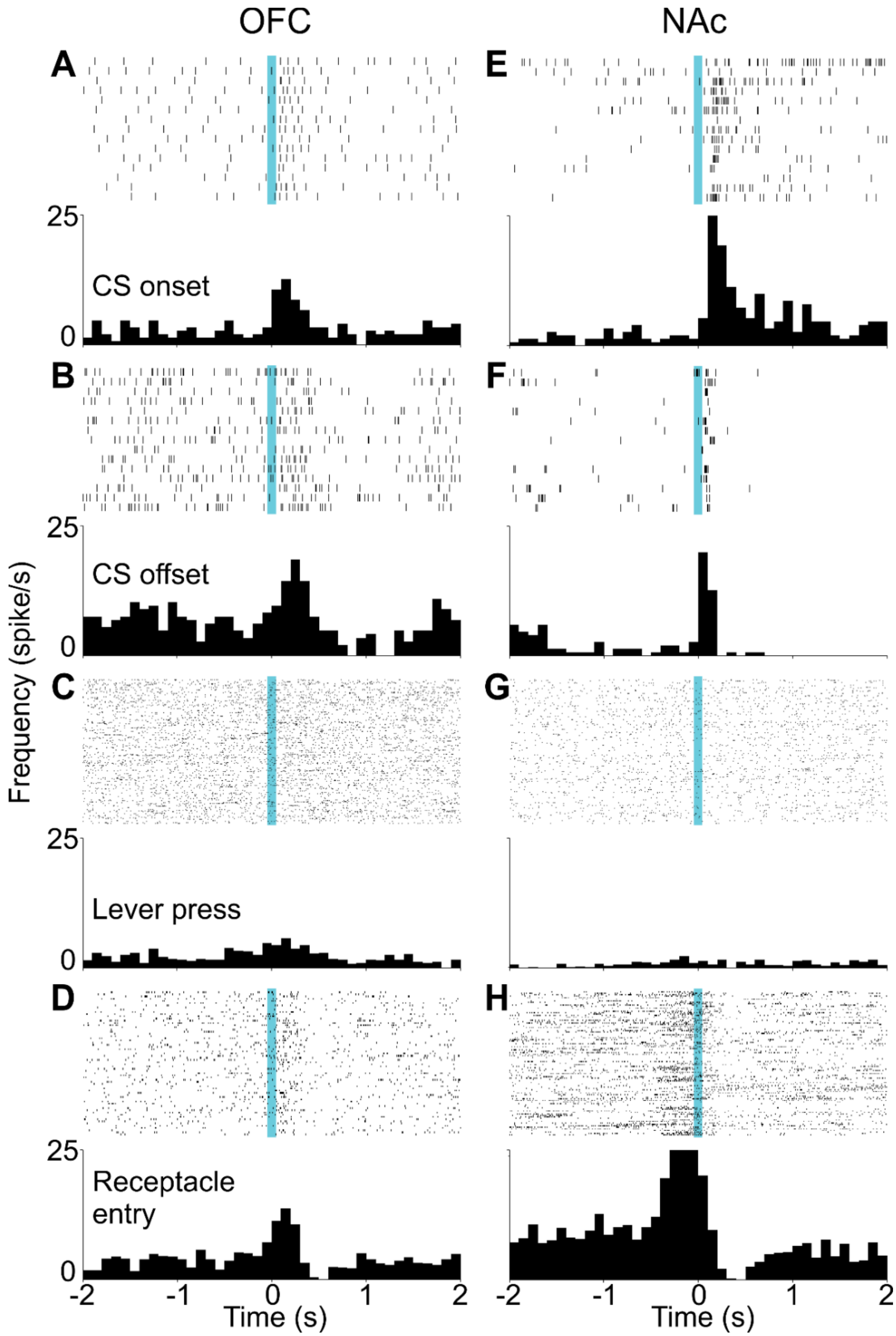

Supplemental Figure S4. Examples of individual neuron activity. A – H: Neuronal activity during events of interest (blue bar) is presented in rasters and perievent histograms for individual neurons (each panel depicts a different neuron). A 2-second (s) window before and after the events of interest was used for neuronal activity representation.

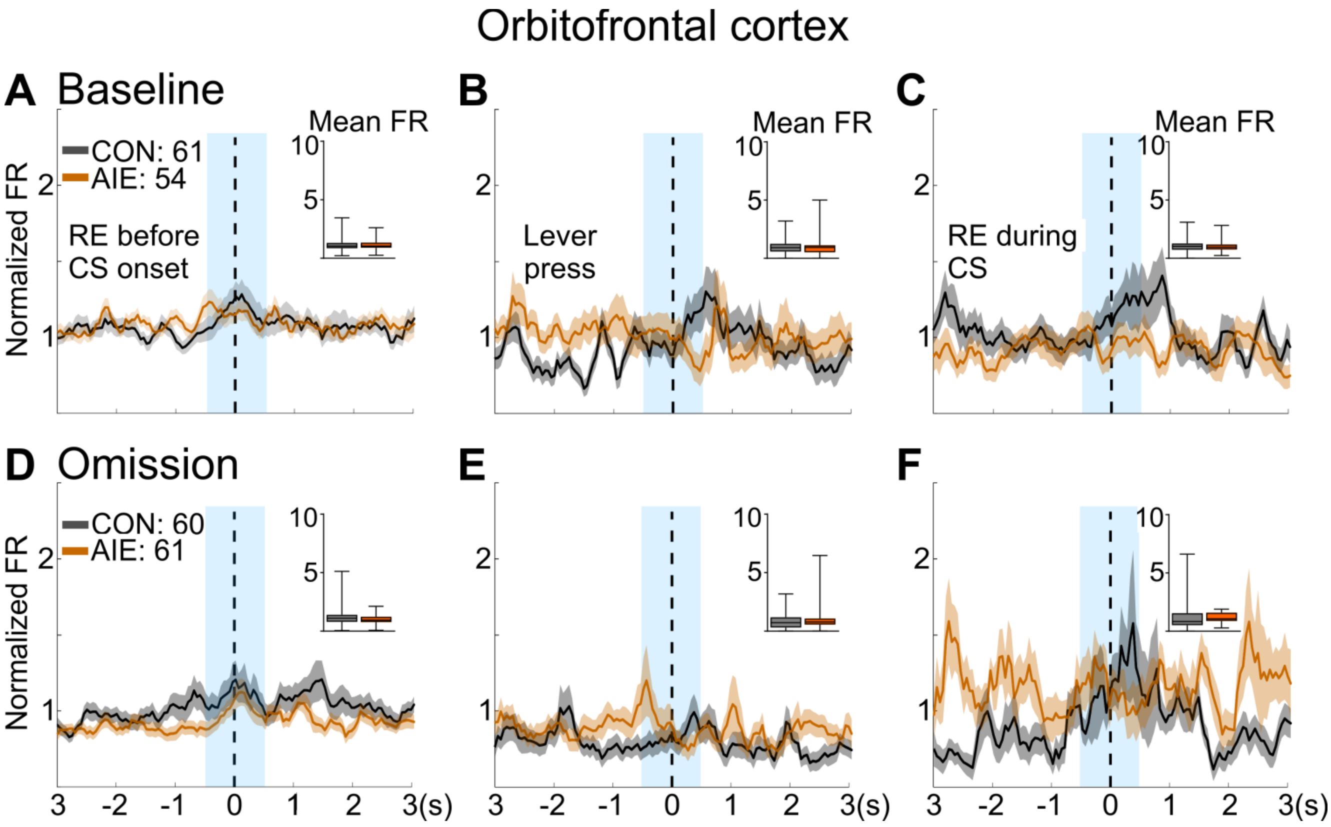

Supplemental Figure S5. No AIE effect was observed on OFC population activity surrounding receptacle entries before CS presentation, first lever press, or first conditioned receptacle entry. Single-unit activity of OFC neurons was analyzed during behavioral responses for the baseline and omission sessions in sign-tracking rats. OFC population activity during the baseline session is presented for the first receptacle entry (RE) 30 s before CS onset (**A**), first lever press (**B**), and first receptacle entry during the 30-s CS period (**C**). No AIE effect was observed on OFC activity recorded during the baseline session. OFC population activity is presented for the first receptacle entry 30 s before CS onset (**D**), first lever press (**E**), and first receptacle entry during the 30-s CS period (**F**). No AIE effect was observed on OFC population activity during behavioral responses after changes in CS-US contingency. Neuronal population activity data was normalized to the whole session firing rate and presented as mean firing rate (line) ± SEM (shading) for CON (gray) and AIE (orange) groups. Number of OFC cells recorded during PCA baseline: CON group = 61 cells and AIE group = 54 cells. Number of OFC cells recorded during omission session: CON group = 60 cells and AIE group = 61 cells. As sign-trackers primarily interact with the CS/lever after CS onset, not all sign-trackers performed receptacle entries during the 30-s CS in all 15 trials. For this reason, only neurons recorded in sign-trackers that performed receptacle entries during the CS in at least 3 trials were analyzed (Baseline: CON= 50, AIE= 44 neurons; Omission: CON= 22, AIE= 37 neurons). Inset: box plots show the mean firing rate in the target window (blue box). These data are presented as median, interquartile range, and minimum and maximum data values. For statistical details see Supplemental Table S2.

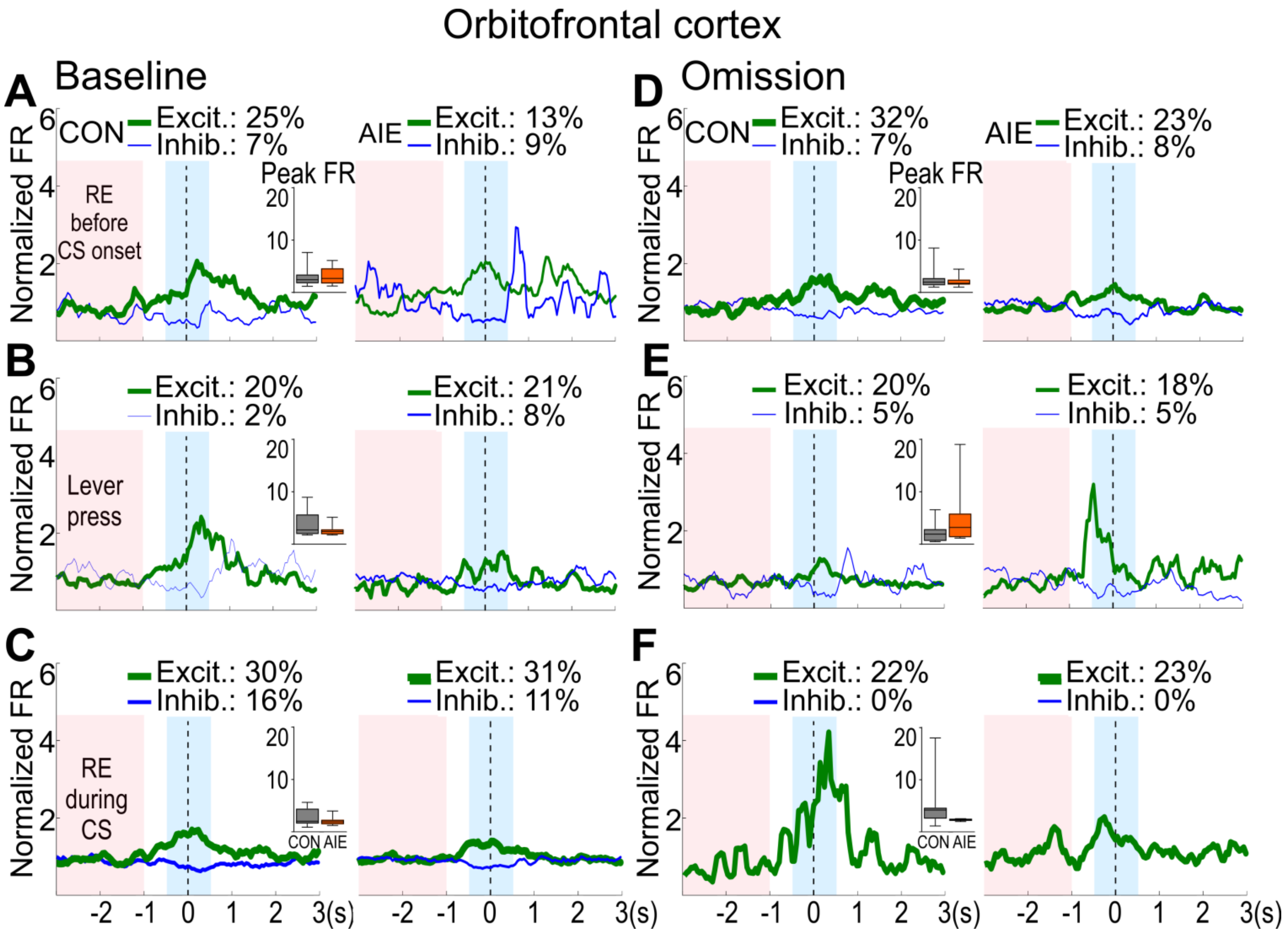

Supplemental Figure S6. No AIE effect was observed on OFC phasic activity during behavioral responses. OFC phasic activity was analyzed during the first receptacle entry 30 s before CS onset, first lever press, and first receptacle entry during the 30-s CS period during baseline and omission sessions. Firing activity for neurons that showed phasic excitatory activity (in green) and phasic inhibitory activity (in blue) is shown in each figure for CON (**left graphs**) and AIE (**right graphs**). The red box represents the 2 s baseline window for the phasic activity analysis. The thickness of each line reflects the proportion of neurons exhibiting phasic excitation or inhibition (non-phasic cells are not shown). Inset: box plots show the amplitude of excitation during the target window (blue box) for each phasic excitatory cell. OFC phasic activity during baseline session is presented for the first receptacle entry (RE) 30 s before CS onset (**A**), first lever press (**B**), and first receptacle entry during the 30-s CS period (**C**). Phasic activity during omission session is presented for the first receptacle entry 30 s before CS onset (**D**), first lever press (**E**), and first receptacle entry during the 30-s CS period (**F**). Both groups showed a similar percentage of phasic excitatory activity surrounding behavioral responses during both sessions. No group difference was observed in the amplitude of the activity displayed by phasic excitatory cells during these events during both sessions. Neuronal phasic activity data are presented as mean firing rate. Box plots showed the median, interquartile range, and minimum and maximum data values. For statistical details see Supplemental Table S2.

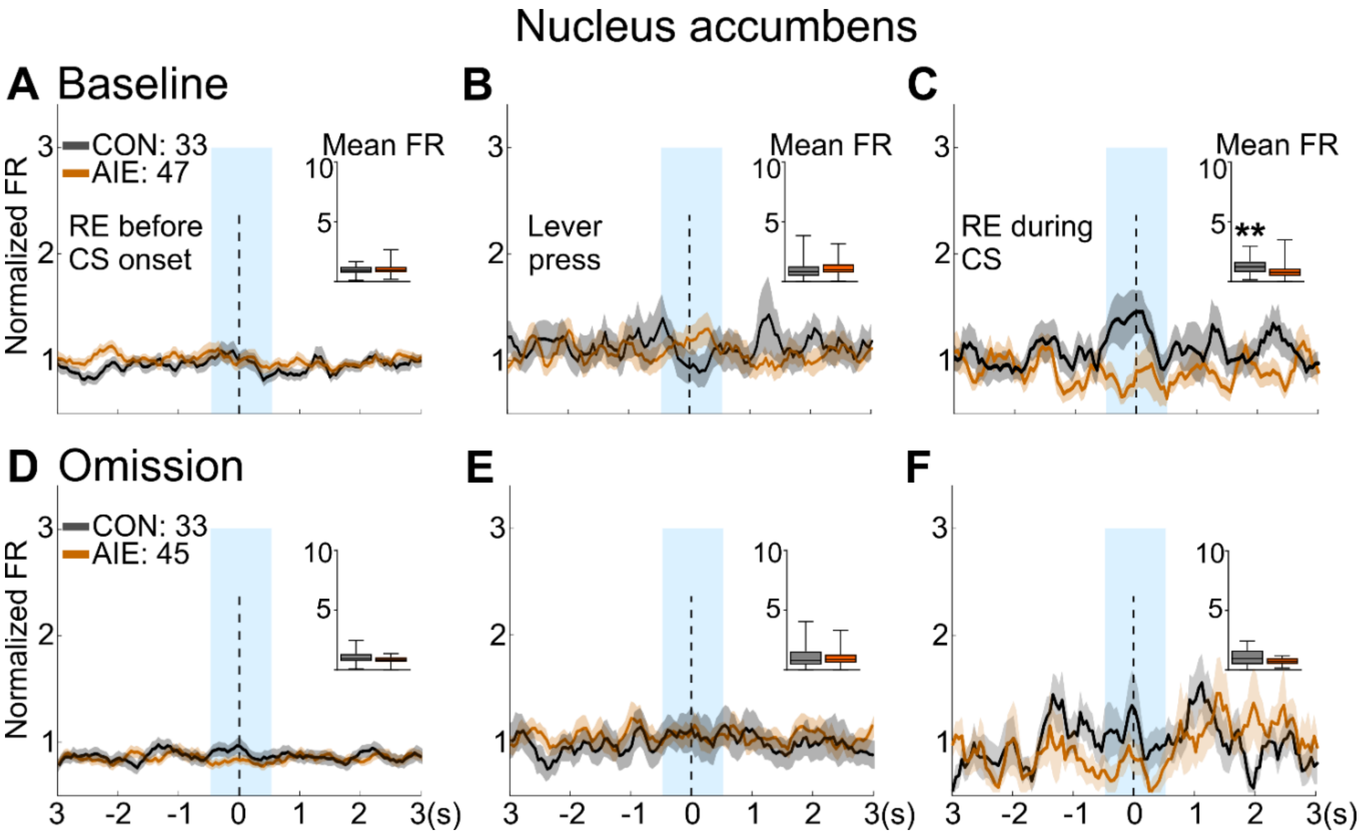

Supplemental Figure S7. NAc neuronal population activity recorded from CON-exposed rats was increased surrounding conditioned receptacle entries during the baseline session. Single-unit activity of NAc neurons was analyzed during the first receptacle entry (RE) 30 s before CS onset, first lever press, and the first receptacle entry during the 30-s CS period performed in each trial of baseline and omission sessions. **A**. No effect of AIE was observed on NAc activity during unconditioned receptacle entries during baseline sessions. **B**. No effect of AIE was observed on NAc activity during the first lever press. **C**. NAc neuronal population activity was significantly higher around conditioned receptacle entries. **D.** No effect of AIE was observed on NAc activity surrounding unconditioned receptacle entries during the omission session. **E.** No group difference was observed on NAc neuronal activity during the first lever press during the omission session. **F.** Also, no difference was observed on the conditioned receptacle entry. Neuronal population activity data are normalized to the whole session firing rate and presented as mean firing rate (line) ± SEM (shading) for CON (gray) and AIE (orange) groups. Number of NAc cells recorded during PCA baseline: CON group = 33 cells on CON AIE group = 47 cells. Number of NAc cells recorded during the omission session: CON group = 33 cells and AIE group = 45 cells. As sign-trackers primarily interact with the CS/lever after CS onset, not all sign-trackers performed receptacle entries during the 30-s CS in all 15 trials. For this reason, only neurons recorded in sign-trackers that performed receptacle entries during the CS in at least 3 trials were analyzed (Baseline: CON= 24, AIE= 34 neurons; Omission: CON= 16, AIE= 13 neurons). Inset: box plots show the mean firing rate in the target window (blue box). ** Significant group difference (P<0.01). For statistical details see Supplemental Table S2.

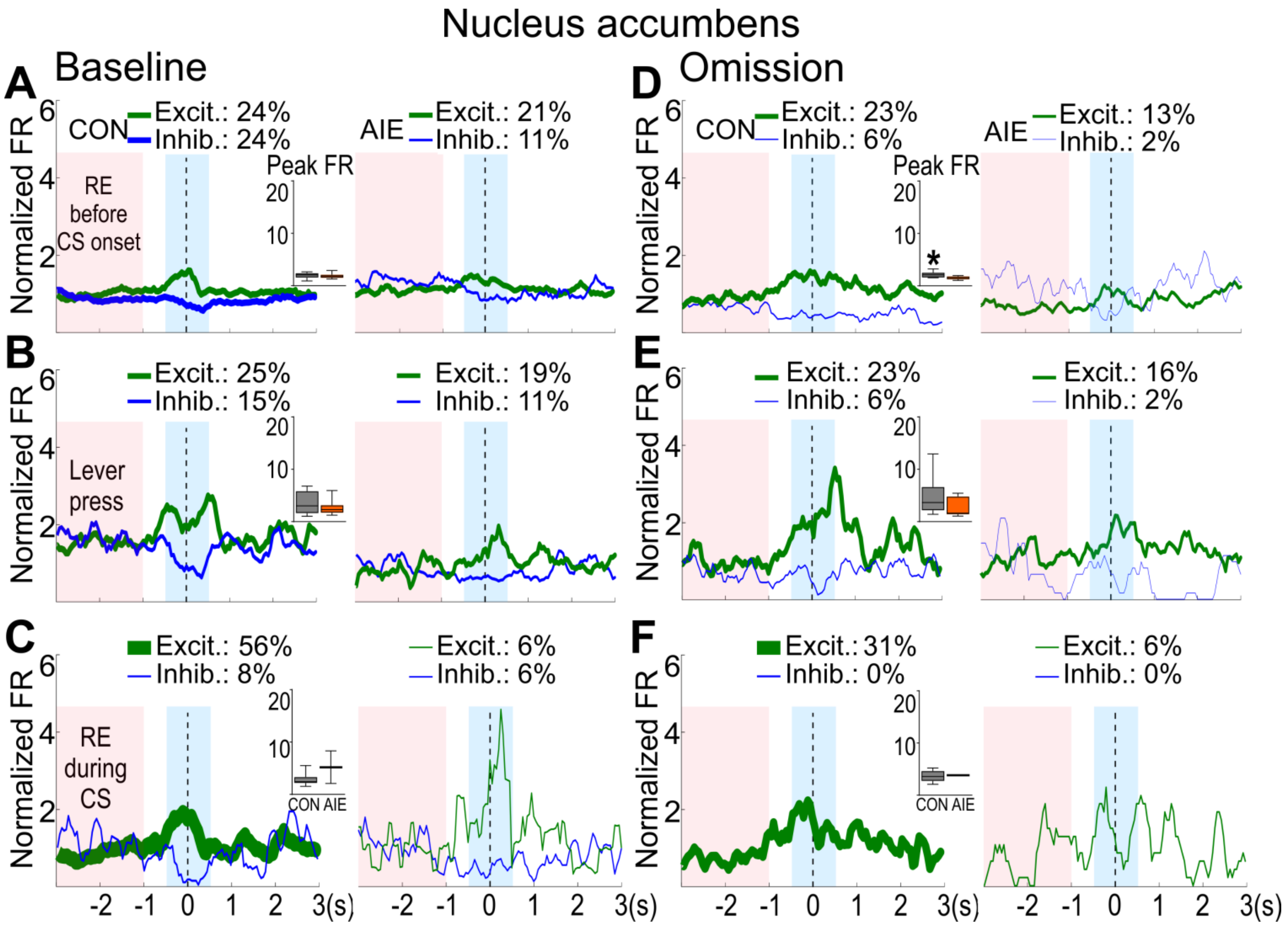

Supplemental Figure S8. A higher percentage of neurons recorded from the NAc of CON-exposed rats showed phasic excitatory activity during conditioned receptacle entries during the baseline session. Phasic activity from NAc neurons was analyzed during PCA baseline and omission sessions. Firing activity for neurons that showed phasic excitatory activity (in green) and phasic inhibitory activity (in blue) is shown in each figure for CON (**left graphs**) and AIE (**right graphs**). The red box represents the 2-s baseline window for the phasic activity analysis. The thickness of each line reflects the proportion of neurons exhibiting phasic excitation or inhibition (non-phasic cells are not shown). Inset: box plots show the amplitude of excitation during the target window (blue box) for each phasic excitatory cell. NAc phasic activity during PCA baseline (**A**, **B,** and **C**) and omission session (**D**, **E,** and **F**) is presented surrounding the first receptacle entry (RE) 30 s before CS onset, first lever press, and first receptacle entry during the 30-s CS period, respectively. **A**. Both groups presented a similar percentage of phasic activity during unconditioned receptacle entries. No AIE effect was observed on the amplitude of the activity displayed by phasic excitatory cells. **B**. No group difference was observed on the percentage or amplitude of the phasic excitatory activity during the first lever press. **C**. A higher percentage of NAc neurons recorded from the CON-exposed group displayed phasic excitatory activity during conditioned entries. No effect was observed on the amplitude of the phasic excitatory activity. **D**. During the omission session, NAc neurons recorded from CON-exposed group displayed a higher amplitude of phasic excitation compared to the AIE group during unconditioned entries. **E.** No group difference was observed in NAc activity surrounding the first lever press. **F.** No group difference was observed during conditioned entries. * Significant group difference in the amplitude of phasic excitatory activity ($P \leq 0.05$). Neuronal phasic activity data are presented as mean firing rate. Box plots showed the median, interquartile range, and minimum and maximum data values. For statistical details see Supplemental Table S2.

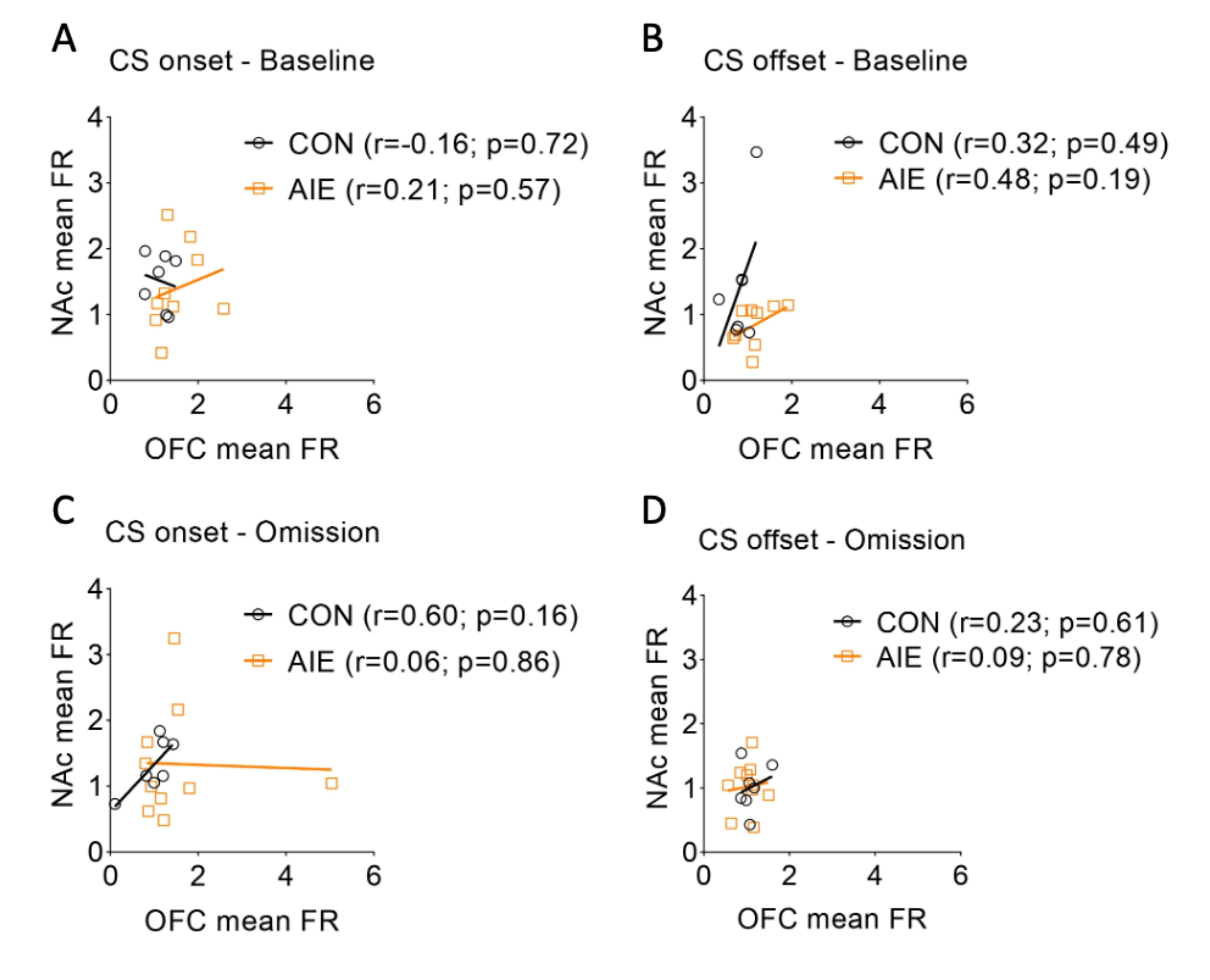

Supplemental Figure S9. No significant correlation was observed between OFC and NAc firing activity for CS onset and CS offset. A-B. Correlation of the mean firing rate (FR) on OFC and NAc in response to CS onset (**A**) and CS offset (**B**) during the baseline session. **C-D.** Correlation of the mean firing rate (FR) on OFC and NAc in response to CS onset (**C**) and CS offset (**D**) during the omission session. The mean neuronal firing activity 1 second after the CS onset or CS offset was averaged in each rat. Only rats with neurons recorded from both OFC and NAc were included in this analysis. P>0.05 after Spearman's correlation analysis.

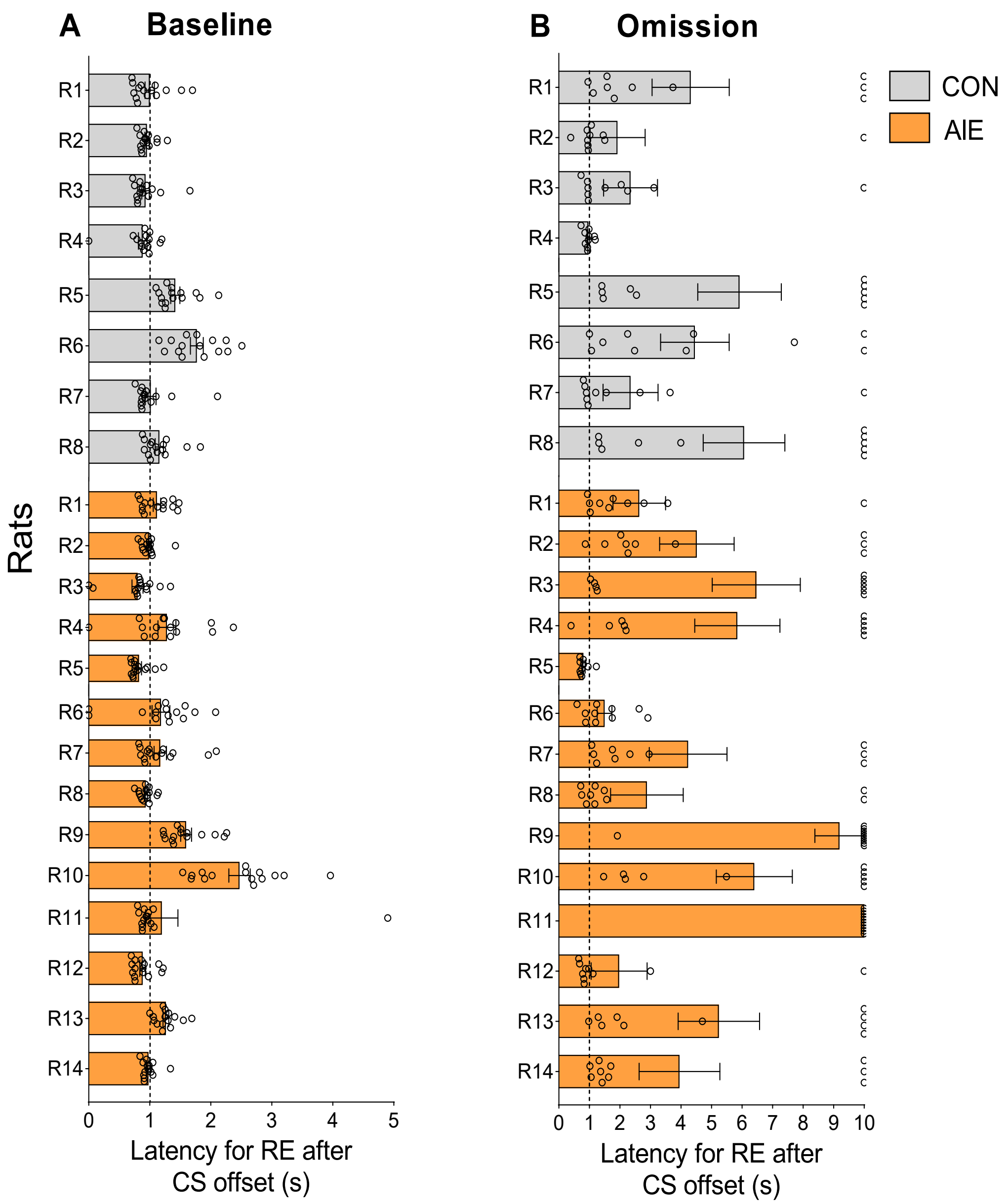

Supplemental Figure S10. Latency for the first receptacle entry after CS offset per rat. The latency for the first receptacle entry (RE) after CS offset is represented for individual control-exposed (CON) and ethanol-exposed (AIE) rats during the baseline (**A**) and omission session (**B**). Data are expressed as mean ± SEM. The symbols represent the latency for RE in individual trial. Dotted line represents the 1 s after CS offset (the analysis window for the single-units electrophysiological data analysis).
